# Sadness-Based Approach-Avoidance Modification Training for Subjective Stress in Adults: Pilot Randomized Controlled Trial

**DOI:** 10.2196/50324

**Published:** 2023-11-30

**Authors:** Lydia Helene Rupp, Marie Keinert, Stephanie Böhme, Lena Schindler-Gmelch, Bjoern Eskofier, Björn Schuller, Matthias Berking

**Affiliations:** 1 Department of Clinical Psychology and Psychotherapy Friedrich-Alexander-Universität Erlangen-Nürnberg Erlangen Germany; 2 Department of Clinical Psychology and Psychotherapy Technische Universität Chemnitz Chemnitz Germany; 3 Machine Learning and Data Analytics Lab, Department Artificial Intelligence in Biomedical Engineering Friedrich-Alexander-Universität Erlangen-Nürnberg Erlangen Germany; 4 Chair of Embedded Intelligence for Health Care and Wellbeing University of Augsburg Augsburg Germany; 5 Group on Language, Audio, & Music Imperial College London London United Kingdom

**Keywords:** stress, emotion, eHealth, approach-avoidance, mental health, somatic health, chronic stress, intervention, stress-related illness, app-based, stress management, belief, training, mobile phone

## Abstract

**Background:**

A key vulnerability factor in mental health problems is chronic stress. There is a need for easy-to-disseminate and effective interventions to advance the prevention of stress-related illnesses. App-based stress management trainings can fulfill this need. As subjectively experienced stress may be influenced by dysfunctional beliefs, modifying their evaluations might reduce subjective stress. Approach-avoidance modification trainings (AAMT) can be used to modify stimulus evaluations and are promising candidates for a mobile stress intervention. As the standard training reactions of the AAMT (swiping and joystick motion) have little valence, emotions could be incorporated as approach and avoidance reactions to enhance the effectiveness of AAMTs.

**Objective:**

We aimed to evaluate the feasibility of a mobile emotion-enhanced AAMT that engages users to display sadness to move stress-enhancing beliefs away and display positive emotions to move stress-reducing beliefs toward themselves (emotion-based AAMT using sadness and positive emotions [eAAMT-SP]). We explored the clinical efficacy of this novel intervention.

**Methods:**

We allocated 30 adult individuals with elevated stress randomly to 1 of 3 conditions (eAAMT-SP, a swipe control condition, and an inactive control condition). We evaluated the feasibility of the intervention (technical problems, adherence, usability, and acceptability). To explore the clinical efficacy of the intervention, we compared pretest-posttest differences in perceived stress (primary clinical outcome) and 3 secondary clinical outcomes (agreement with and perceived helpfulness of dysfunctional beliefs, emotion regulation, and depressive symptoms) among the conditions.

**Results:**

The predetermined benchmarks of 50% for intervention completion and 75% for feasibility of the study design (completion of the study design) were met, whereas the cutoff for technical feasibility of the study design (95% of trials without technical errors) was not met. Effect sizes for usability and acceptability were in favor of the eAAMT-SP condition (compared with the swipe control condition; *intelligibility of the instructions*: *g*=−0.86, *distancing from dysfunctional beliefs*: *g*=0.22, and *approaching functional beliefs*: *g*=0.55). Regarding clinical efficacy, the pretest-posttest effect sizes for changes in perceived stress were *g*=0.80 for the comparison between the eAAMT-SP and inactive control conditions and *g*=0.76 for the comparison between the eAAMT-SP and swipe control conditions. Effect sizes for the secondary clinical outcomes indicated greater pretest-posttest changes in the eAAMT-SP condition than in the inactive control condition and comparable changes in the swipe control condition.

**Conclusions:**

The findings regarding the feasibility of the intervention were satisfactory except for the technical feasibility of the intervention, which should be improved. The effect sizes for the clinical outcomes provide preliminary evidence for the therapeutic potential of the intervention. The findings suggest that extending the AAMT paradigm through the use of emotions may increase its efficacy. Future research should evaluate the eAAMT-SP in sufficiently powered randomized controlled trials.

**Trial Registration:**

German Clinical Trials Registry DRKS00023007; https://drks.de/search/en/trial/DRKS00023007

## Introduction

### Background

A delayed train, an impending deadline, or financial hardships—these are examples of situations that may pose significant demands on an individual and, hence, induce stress. Experiencing acute stress in such situations does not necessarily affect health, but chronic stress is associated with somatic [1,2] and mental health problems [3-5]. As chronic stress affects up to 33% of the adult population [6,7], there is a significant need for low-threshold and accessible interventions for effective stress management.

It is of note that the presence of an objective stressor alone does not determine the subjective stress response. Instead, the latter is shaped to a large extent by individuals’ subjective assessment of such stressors as threats to personal goals as well as the assessment of available coping resources [8-12]. As such, these assessments are likely to be influenced by subjective beliefs that affect (1) the salience of goals, (2) the perceived discrepancy between the current situation and salient goals, or (3) the assessment of one’s ability to diminish the corresponding discrepancies. Such beliefs can be automatic or reflective in nature [13] and can be considered *dysfunctional* if they promote an unhelpful intensity or duration of stress [14,15].

On the basis of this cognitive model of stress, many stress management interventions systematically target dysfunctional beliefs through cognitive behavioral interventions [16-20]. Although a large number of studies provide evidence for the efficacy of such cognitive treatments [19,20], these interventions (such as the Socratic dialogue [21]) rely heavily on reflective processes that require higher-order cognitive processing capacities that, however, have been found to be impaired under stress [22-26]. Thus, it can be hypothesized that stress interventions would benefit from diminishing dependency on a resource that is limited when it is most needed. Instead, stress management interventions should increase their focus on automatic or associative processes that require less higher-order processing capacities [27], such as the modification of dysfunctional associative structures [28,29].

An evidence-based approach to modify automatic stimulus evaluation tendencies is approach-avoidance modification training (AAMT) [30,31]. Commonly, AAMTs present stimuli cueing either functional or dysfunctional behavior on a computer screen (eg, alcoholic vs nonalcoholic beverages for individuals with alcohol use disorder) and require participants to use pull and push movements with a joystick to move dysfunctional stimuli away from themselves and functional stimuli toward themselves [32]. Arguably, the repeated approach and avoidance of training stimuli initiates an inferential process that results in a modification of the subjective stimulus evaluation [33]. By pushing a stimulus away, the valence associated with this avoidance reaction (ie, “This is something I have to avoid”) is assumed to become associated with said stimulus. As a result, the stimulus should be evaluated more negatively. For complex stimuli requiring more conscious cognitive processing (such as written statements), cognitive dissonance [34] might be a second working mechanism of AAMTs—pulling a functional belief toward oneself might generate cognitive dissonance if the belief is not shared by the participant. As participants continuously have to pull functional beliefs toward themselves, they might experience a dissonance-driven pressure to share the functional belief. Similarly, pushing away beliefs that are dysfunctional but shared by the participant might generate dissonance and, hence, motivate participants to distance themselves from the dysfunctional belief. Thus, it can be hypothesized that AAMTs can be used to modify evaluations at both an implicit and explicit level.

Empirically, the efficacy of AAMTs has been demonstrated in various clinical applications. Significant effects on clinical outcomes have been found for alcohol use disorder [35,36], depressive symptoms [37,38], social anxiety disorder [39,40], cannabis use [41], body dissatisfaction and other symptoms of eating disorders [42,43], and procrastination [44]. However, some studies have found effects only for automatic approach-avoidance tendencies but not for clinical outcomes [45-47]. In the context of stress, 3 studies reported in 2 papers have evaluated the effect of AAMTs on subjective stress after a stress-inducing task. The studies trained participants to approach and avoid *positive* and *negative* emotional, but not directly stress-related pictures from affective picture data bases (eg, humans, animals, and objects) with the goal of fostering the approach tendencies toward positive stimuli [48,49]. Although these studies further demonstrated the efficacy of AAMTs in modifying approach-avoidance tendencies, findings regarding effects on stress are limited as an effect was only found in one subgroup of individuals with dysphoria in the second study by Becker et al [48]. In the study by Ferrari et al [49], no effects on stress reactivity (assessed as changes in heart rate variability before and after a stress-inducing task) were found.

Building on these studies, we propose that AAMTs targeting subjective stress may be more effective if they explicitly address stress-related beliefs (instead of pictures of positive or negative stimuli). In addition, the efficacy of AAMTs might be further enhanced by interacting with the stimuli in a way that carries more valence than simple wrist or hand movements. As the conscious display of emotions can be assumed to carry a valence much stronger than that of a small wrist or hand motion [50], it can be hypothesized that stress-causing beliefs can be effectively modified by AAMTs using positive emotions to pull functional beliefs toward oneself and negative emotions to push dysfunctional beliefs away from oneself. Moreover, it is of note that emotions embody specific meanings and facilitate certain action tendencies [51-53] that might further increase the efficacy of the intervention by providing additional information in the inferential process aimed to modify stimulus evaluations [33].

An emotion that arguably holds particular promise for emotion-based AAMTs (eAAMTs) is sadness. This emotion is central, commonly experienced [54], and typically associated with a negative valence [55], which, hence, may cue avoidance [56]. Importantly, experiencing sadness facilitates disengagement from unattainable goals [55,57-59]. Moreover, it is associated with typical facial expressions, vocal tone, and body posture that have been shown to cue sadness when purposefully enacted [60]. With regard to strengthening stress-reducing beliefs, positive emotions may be used in the same fashion. Emotions such as joy, satisfaction, pride, or love carry strong positive valence and are associated with approach tendencies [61] as well as typical facial and bodily expressions.

Thus, it might be possible to use sadness and positive emotions in an eAAMT (eAAMT-SP) to increase the effectiveness of the avoidance response by displaying sadness to distance oneself from stimuli representing dysfunctional beliefs. It can further be hypothesized that, by doing so, negative valence and avoidance tendencies inherent in sadness influence the inferential process, leading to a more negative evaluation and avoidance of the dysfunctional beliefs. Finally, the potential of sadness to facilitate disengagement may also foster disengagement from dysfunctional beliefs (and their reinforcing characteristics, similar to dysfunctional beliefs in insomnia) [62]. Similarly, pulling functional beliefs toward oneself by enacting (and, hence, to some extent, experiencing) positive emotions should transfer positive valence and approach motivation toward these stimuli and, potentially, to the beliefs themselves. In addition, systematically inducing positive emotions should, by itself, interfere with prolonged stress experiences and level off stress-related allostatic load [63,64].

### Objectives

Despite these arguments for the potential of using emotions in AAMTs, such an approach has not yet been empirically investigated. Thus, the primary goal of this study was to explore the feasibility of an innovative smartphone-based eAAMT that uses expressions of sadness to foster avoidance of stress-enhancing beliefs and expressions of positive emotions to foster an approach toward stress-reducing beliefs. Second, this study explored the clinical efficacy of the novel intervention with regard to perceived stress and agreement with and perceived helpfulness of dysfunctional beliefs, emotion regulation, and depressive symptoms.

## Methods

### Design

We report the results of an 8-armed randomized controlled trial that aimed to evaluate the feasibility and explore the clinical efficacy of several variations of the new eAAMT paradigm in the context of elevated stress (N=82). In this paper, we report the effects of 3 of the study arms, namely, the eAAMT-SP when compared with 2 control conditions (a swipe control condition and an inactive control condition) with regard to feasibility and effects on experienced stress level, the evaluation of stress-related beliefs, emotion regulation skills, and depression. A detailed description of the entire study can be found in the protocol paper [65]. The findings for the other eAAMT conditions will be reported elsewhere.

### Participants and Procedure

**Figure 1:**
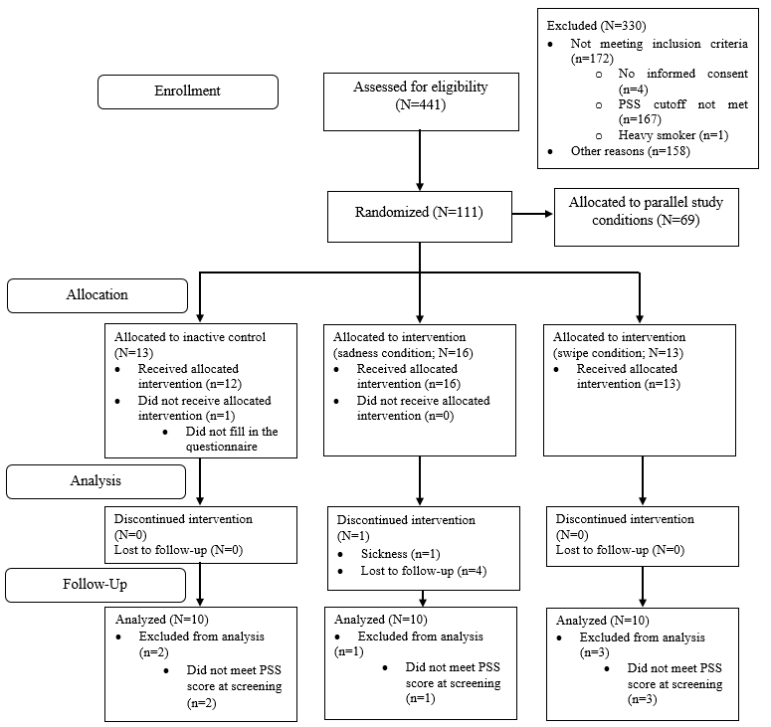
CONSORT (Consolidated Standards of Reporting Trials) chart of participant flow from recruitment to follow-up assessment. PSS: Perceived Stress Scale.

Recruitment took place in Erlangen, Germany, via posts on social networks, email newsletters of the Friedrich-Alexander-Universität Erlangen-Nürnberg, and flyers posted in public places between July 2020 and May 2022. Psychology students received course credit in compensation for their participation. Inclusion and exclusion criteria were assessed with the help of the web-based questionnaire tool Questback [66]. Participants were included in the study if they (1) reported elevated perceived stress as indicated by a score of ≥19 on the 10-item version of the Perceived Stress Scale (PSS-10) [67], (2) were aged ≥18 years, and (3) provided informed consent for study participation. Exclusion criteria were (1) the presence of psychotic disorders, (2) physical impairments in emotion expression (eg, facial paralysis), (3) heavy smoking (ie, >10 cigarettes per day; owing to the parallel collection of cortisol samples, which is not reported in this paper because of a deviating focus of this manuscript [65,68]), and (4) current suicidal ideation. A cutoff score of 19 on the PSS-10 was defined as 1 SD above the mean score in the German version of the PSS-10 [67]. This was implemented according to the suggested procedures by Heber et al [69]. As recommended by Whitehead et al [70], we chose a sample size of 10 for each treatment arm. Eligible participants were randomly allocated to 1 of the 3 study conditions (eAAMT-SP, swipe control group, and inactive control group). For this purpose, we used block randomization (according to random numbers generated in Excel [Microsoft Corp]) with a block size of 8. Randomization was conducted by a research assistant who was otherwise not involved in the study. An overview of the participant flow is shown in Figure 1.

### Ethical Considerations

The study was conducted in accordance with ethical standards as defined in the Declaration of Helsinki and was approved by the German Psychological Societies ethics committee (BerkingMatthias2020-09-10AM). Participants provided written informed consent before filling out the web-based screening questionnaire. Study data were deidentified for analysis and stored separately from nonanonymous participant data (eg, contact information necessary for follow-up assessments). Compensation for study participation included either monetary compensation or course credit. Psychology students could receive course credit for their participation. All participants had the option to enter a draw and win €500 (US $530.80; until May 2021) or receive an expense allowance of €20 (US $21.23; from June 2021 onward; from then on, psychology students could choose between financial compensation and course credit). This change in the recruitment procedure was made because of difficulties in recruitment speed and reaching the target sample size.

### Study Conditions

The study included 3 conditions. The eAAMT-SP condition served as the experimental condition. We included 2 control conditions, one active control group that received a standard swipe AAMT and an inactive control group that received no training.

In the eAAMT-SP and swipe control conditions, participants completed a smartphone-based training on 4 consecutive days. The training was conducted by research assistants (8 bachelor’s students, 4 master’s students, and 1 doctoral candidate) in psychology who were trained and supervised by a clinical psychologist with a master’s degree in psychology. All experimenters followed a manual that detailed the procedure of the training sessions. The daily training period lasted between 20 and 30 minutes. During the training, participants were confronted with stress-enhancing (eg, “If I fail partly, it is as bad as a complete failure”) and stress-reducing (eg, “I am allowed to make mistakes”) beliefs. The beliefs were presented as written statements accompanied by illustrations on the smartphone screen. For each training session, 60 beliefs were randomly selected from a pool of 78 beliefs (30 stress-enhancing beliefs and 30 stress-reducing beliefs randomly selected from a pool of 48 beliefs) with a 50:50 ratio for the presentation of stress-reducing and stress-enhancing beliefs. The pool of 78 stress-related beliefs was based on the Dysfunctional Attitude Scale (DAS) [71] and supplemented by additional stress-relevant beliefs developed by a group of experts in clinical psychology. Stress-reducing and stress-enhancing beliefs were presented in a random order during each training session. Participants were instructed to perform an approach response when presented with a stress-reducing belief and to perform an avoidance response when presented with a stress-enhancing belief.

In the eAAMT-SP condition, participants were instructed to display sadness in response to stress-enhancing beliefs. More specifically, they were invited to display a sad facial expression, take a deep breath, tilt their head forward, and say “I will let go of this belief.” In response to stress-reducing beliefs, participants were instructed to display joy, relaxation, or love on the first day of training; excitement, tranquility, or gratitude on the second day; happiness, resolve, or contentment on the third day; and courage, confidence, or pride on the last day of training. Similar to the sadness instructions, they were instructed to display the facial expression of the corresponding emotion, enact a behavioral expression consistent with the emotion (eg, a raised fist for pride), and utter a verbal statement also consistent with the emotion (eg, “Yes, I did it!” for pride). Instructions on the responses were first provided by the experimenter and then through text and a video on the training app. Participants completed 2 practice trials together with the experimenter and were provided feedback on whether they had shown the correct response. During the training, the experimenter monitored the participants’ responses and emotional expressions through a video feed and triggered movements of the stimuli on the smartphone screen via a remote control if the emotion was shown as instructed (similar to a Wizard of Oz paradigm [72] but modified in the sense that participants always knew that the stimuli were moved by the experimenter).

In the swipe control condition, participants were instructed to swipe stress-enhancing beliefs away from themselves (from bottom to top) and stress-reducing beliefs toward themselves (from top to bottom) on the smartphone screen [65]. In both the eAAMT-SP and swipe control conditions, the stimulus became smaller if participants showed sadness (eAAMT-SP condition) or moved it away from themselves (swipe control condition), thereby creating an illusion of increased spatial distance. Correspondingly, the stimulus became larger to create an illusion of reduced spatial distance if participants showed positive emotions (eAAMT-SP condition) or pulled the stimulus toward themselves (swipe control condition). If the correct response was given by the participant, a thumbs-up picture was displayed to provide reinforcing feedback. If the displayed emotion was not correct, participants received the following feedback—“Unfortunately, this was the wrong response”—on the smartphone screen. Participants in the inactive control condition received the outcome questionnaires via email without participating in any kind of intervention.

Participants who were allocated to either the eAAMT-SP (experimental condition) or the swipe control condition were blinded to whether they had received the experimental intervention or the active control intervention. Participants who were allocated to the inactive control group were aware that they had not received any intervention. The study personnel delivering the interventions were not blinded to the group allocation of the participants.

### Measures

Outcome measures were assessed at 3 time points. The first assessment (preassessment time point; T1) was conducted immediately before the first training session, the second assessment (T2) was conducted after the last (ie, fourth) training session, and the third assessment (postassessment time point; T3) was conducted 1 week after T2. For all assessments, questionnaires were administered via the web-based survey tool Unipark [66].

### Feasibility

The primary outcome was the feasibility of the novel eAAMT-SP. To evaluate the feasibility, we used methods proposed within the process evaluation framework [73] and evaluated technical problems, adherence, usability, and acceptability of the intervention. Technical problems were evaluated by assessing whether ≥95% of the study trials were completed without technical difficulties that would lead to termination or interruption of or delays of >5 minutes in the study trial. Adherence to the intervention was defined as ≥50% of the participants completing the intervention as instructed. To assess the feasibility of the study design, the number of eligible study participants, the percentage of those willing to participate in the study, and the number of completed follow-up assessments were analyzed. The study design would be considered feasible if at least 75% of the follow-up assessments were completed correctly. Usability of the intervention was assessed with the help of 4 self-constructed usability questions that were administered after the last training session. Participants in the eAAMT-SP and swipe control conditions rated (1) the intelligibility of the instructions presented within the app (0=*very difficult*; 5=*very easy*), (2) the perceived ease with which they displayed the emotions at the beginning versus at the end of the training (0=*very difficult*; 5=*very easy*), (3) how much they were able to distance themselves from dysfunctional beliefs through the training (0=*not at all*; 5=*completely*), and (4) how much they were able to approach functional beliefs through the training (0=*not at all*; 5=*completely*). Acceptability was assessed using 2 additional self-constructed items. Participants in the eAAMT-SP and swipe control conditions were asked to evaluate (1) the usefulness of the training with regard to the modification of stress-related beliefs (0=*not at all useful*; 5=*very useful*) and (2) whether they would recommend the training to a friend (0=*very unlikely*; 5=*very likely*).

### Clinical Outcomes

The secondary outcomes of this study included 4 clinical variables. The primary clinical outcome in this study was the *subjective stress level* as assessed using the 10-item German version of the PSS-10 [67]. This questionnaire measures feelings of unpredictability and uncontrollability in daily life within the previous week. Items are rated on a 5-point Likert scale (0=*never*; 4=*very often*). The scale was found to have good validity and reliability (Cronbach α=.84) [67]. In this study, the Cronbach α was .85 (95% CI 0.68-0.92) at T1 and .88 (95% CI 0.74-0.93) at T3. The correlation of the PSS-10 scores between T1 and T3 was *r*=0.55.

In addition to the primary clinical outcome, 3 secondary clinical outcomes were assessed. *Stress-related functional and dysfunctional beliefs* were assessed using 10 selected items from the short version of the DAS [71]. In total, 9 of these items addressed dysfunctional beliefs, whereas the last item assessed a functional belief. The items were selected based on the extent to which they fit the topic of stress by a group of 3 experts in clinical psychology (including the last author). The items selected were items 3, 5, 6, and 7 from the DAS form A (DAS-A) and items 3, 4, 5, 9, 10, and 18 from DAS form B [71]. In addition to the original format of assessing the extent of agreement versus disagreement with these statements (DAS-A; 7-point-Likert scale ranging from 1=*fully disagree* to 7=*fully agree*), we further asked how helpful participants considered their respective beliefs to be (DAS form H [DAS-H]; 7-point-Likert scale ranging from 1=*not at all helpful* to 7=*very helpful*). For each scale, a total score was obtained by reverse coding the functional belief item and calculating the sum score of the 10 items. The original DAS has been found to have good reliability in both clinical and nonclinical populations (Cronbach α=.80-.89) [71]. In this study, the Cronbach α was .80 (95% CI 0.63-0.88) at T1 and .87 (95% CI 0.80-0.90) at T3 for the DAS-A and .85 (95% CI 0.68-0.91) at T1 and .93 (95% CI 0.79-0.97) at T3 for the DAS-H. The correlation of the DAS-A and DAS-H scores between T1 and T3 was *r*=0.74 and *r*=0.63, respectively.

*Emotion regulation skills* were assessed using the Prolonged-State Version of the Emotion Regulation Skills Questionnaire (ERSQ; German version [74]). The 27-item self-report measure assesses the successful application of 9 emotion regulation strategies (awareness, body perception, clarity, understanding, acceptance, resilience, readiness to confront, self-support, and modification) during the previous week on a 5-point Likert scale (0=*not at all*; 4=*almost always* ). The average score across all 27 items is used as a general indicator of the availability of adaptive emotion regulation skills. The questionnaire has been found to have acceptable to good reliability (Cronbach α=.71-.83) [74-79]. In this study, the Cronbach α of the ERSQ was .89 (95% CI 0.74-0.94) at T1 and .90 (95% CI 0.78-0.94) at T3. The correlation of the ERSQ scores between T1 and T3 was *r*=0.50.

*Depressive symptom severity* was assessed using the German version of the Center for Epidemiologic Studies Depression Scale (CES-D) [80]. The 20-item self-report questionnaire assesses the frequency of depressive symptoms during the last 7 days on a 4-point Likert scale (0=*some of the time*; 3=*most of the time*). The CES-D has been found to have good reliability and validity in nonclinical samples (Cronbach α=.89-.92) [81,82]. In this study, the Cronbach α of the CES-D was .84 (95% CI 0.66-0.91) at T1 and .89 (95% CI 0.81-0.93) at T3. The correlation of the CES-D scores between T1 and T3 was *r*=0.66.

Several additional outcomes were assessed but, in the interest of space, are not reported in this paper. These included emotional state assessed using an adapted version of the Positive and Negative Affect Schedule [83] in all groups. In the eAAMT-SP and swipe control conditions, current stress (measured using a paper-and-pencil questionnaire before each training and at T1, T2, and T3 in the inactive control condition) and performance measures (measured as ratings of the emotion display by the experimenter and a self-rating of emotion intensity by the participants and, for a randomly selected subsample, using the System Usability Scale [84]) were assessed. Saliva samples (for the analysis of salivary cortisol levels) were collected before the first and last training sessions from all participants. Finally, heart rate, heart rate variability, and respiration rate were collected before and after the first and last training sessions using radar technology [85] in a subsample of participants. The radar system and the assessment using the System Usability Scale were added to the study protocol after the preregistration owing to novel considerations that arose during the preparation of the study.

### Statistical Analysis

As the focus of this study was the evaluation of the feasibility and clinical efficacy of the eAAMT-SP when completed by participants, we used a per-protocol approach for our analyses and included only participants who had completed the entire assessment (T1, T2, and T3 measurement) in the final analyses. In addition, intention-to-treat (ITT) analyses using imputation for missing data were conducted.

To assess the feasibility of the intervention and the study as a whole, we compared the indicators of feasibility between the eAAMT-SP and swipe control conditions. The feasibility questions were evaluated descriptively and using 2-tailed *t* tests. Means, SDs, and ranges were computed. For the variables “intelligibility of the instructions,” “distancing from dysfunctional beliefs,” “approaching functional beliefs,” “modification of beliefs,” and “recommendation to a friend,” the Hedges *g* was calculated for the comparison of feasibility outcomes between the eAAMT-SP and swipe control conditions at T3. In addition, independent *t* tests were conducted to compare the eAAMT-SP and swipe control conditions. Feasibility data were evaluated for the feasibility sample of 29 participants who had been randomized into either the eAAMT-SP or swipe control conditions (note: this sample also included participants who were randomized but were later excluded from the per-protocol analyses as they did not meet the cutoff for the PSS-10 during screening).

To explore the clinical potential of the eAAMT-SP, we calculated Hedges *g* [86] effect sizes to allow for descriptive comparisons across study conditions and descriptive comparisons of effects in the eAAMT-SP condition with effect sizes reported in studies evaluating other stress management interventions. The Hedges *g* was calculated for the comparison of pretest-posttest difference scores among the 3 groups. As benchmarks for the classification of these effect sizes, we used commonly accepted standards and considered *g*=0.20 as a small effect, *g*=0.50 as a medium effect, and *g*=0.80 as a large effect [87]. The therapeutic potential of the intervention was defined as an effect size of *g*≥0.20 for the primary clinical outcome. To explore whether differences across conditions would meet the criteria for statistical significance in spite of the small sample size, we computed a 95% bootstrap CI (calculated using 1000 iterations) and tested differences across conditions regarding primary and secondary clinical outcomes using 2-way ANOVAs. In these analyses, the study condition was included as the between-factor, and time was included as the within-factor. Owing to a partial overlap in the assessment periods between T1 and T2 for some of the clinical outcome variables, we evaluated the change in the clinical outcomes between T1 and T3. To maximize statistical power, we chose a stepwise approach in these ANOVAs and first compared the eAAMT-SP condition with the inactive control condition. Only if this comparison was significant would we proceed to the second step and compare the eAAMT-SP with the swipe control condition. This rationale is based on significant evidence indicating that a swipe control condition would be superior to an inactive control condition [37,38,43,44]. For the comparison between the eAAMT-SP and inactive control conditions, a 1-tailed test was used (as we expected that the eAAMT-SP approach would be superior to no treatment), and a 2-tailed test was used for the comparison between the eAAMT-SP and swipe control conditions. For the 1-tailed test, the empirical P value is reported in this paper and divided by 2 to assess its 1-tailed significance. The ANOVA was conducted using both a per-protocol approach and the ITT principle. For the latter, missing data were imputed using a multiple-chained equation approach [88] with 20 imputations. Missing values in clinical outcome variables were imputed from the available data of these variables (PSS-10, DAS-A, DAS-H, ERSQ, and CES-D). Owing to the small per-protocol sample size of 30, we chose an ANOVA instead of the hierarchical linear model approach described in the preregistration. Differences in demographic data among the 3 conditions at baseline were analyzed using ANOVAs for continuous variables and Fisher *Z* tests for categorical variables. The means and SDs were calculated. Statistical analyses were conducted using R (version 4.1.1; R Foundation for Statistical Computing) [89]. The Hedges *g* was calculated using the *rstatix* package [90]. The ANOVA was conducted using the *afex* package [91]. Multiple imputation was carried out using the *mice* package [92], and the ANOVA for the ITT analysis was conducted using the *miceadds* package [93].

## Results

### Preliminary Analyses

Descriptive statistics for the sociodemographic variables are presented in Table 1. We assessed participants’ gender, age, education, occupation, marital status, and psychotherapy experience. For university students, study period and field of study were assessed. The demographic variables were analyzed for all participants who were randomized into the eAAMT-SP, swipe control, or inactive control condition. The 3 conditions did not differ significantly in any of the outcome variables at baseline (P>.10 in all cases). No substantial differences were found except for the variable “Former psychotherapy.” Compared with the other conditions, more participants in the eAAMT-SP condition reported ever having received psychotherapeutic treatment.

**Table 1:** Demographic characteristics of the participants in the 3 study conditions (emotion-based approach-avoidance modification training using sadness and positive emotions [eAAMT-SP], swipe control, and inactive control) and P values for between-group comparisons (N=30).

		eAAMT-SP (n=10)	Swipe control (n=10)	Inactive control (n=10)	P value	
**Gender, n (%)**						>.99
	Man	1 (10)	1 (10)	2 (20)		
	Woman	9 (90)	9 (90)	8 (80)		
Age (years), mean (SD)		24.7 (6.68)	24.7 (4.92)	25.5 (4.72)	.93	
**Educational level, n (%)**						.70
	High school diploma	8 (80)	6 (60)	6 (60)		
	University degree or technical university degree	2 (20)	4 (40)	4 (40)		
**Occupation, n (%)**						>.99
	University student	8 (80)	8 (80)	8 (80)		
	Employed	2 (20)	2 (20)	2 (20)		
**Degree level, n (%)**						.24
	Bachelor’s degree	7 (70)	5 (50)	5 (50)		
	Master’s degree	1 (10)	2 (20)	3 (30)		
	Diploma	0 (0)	1 (10)	0 (0)		
**Field of study, n (%)**						.31
	Psychology	7 (70)	5 (50)	6 (60)		
	Other	1 (10)	3 (30)	2 (20)		
**Marital status, n (%)**						>.99
	Unwed	9 (90)	9 (90)	9 (90)		
	Married	1 (10)	1 (10)	1 (10)		
**Former psychotherapy, n (%)**						.09
	Yes	4 (40)	0 (0)	1 (10)		
	No	6 (60)	10 (100)	9 (90)		
**Current psychotherapeutic treatment, n (%)**						>.99
	No	10 (100)	10 (100)	10 (100)		

### Feasibility

Of the participants who met the eligibility criteria, 41.3% (111/269) participated in the study. The sample of 111 participants for the full study was recruited within a period of 23 months. A total of 87.5% (14/16) of trials in the eAAMT-SP condition and 84.62% (11/13) of trials in the swipe control condition were completed without any serious technical difficulties leading to the termination of the trial. Thus, the percentage of trials that were completed without technical problems was below the cutoff of 95%. Within each condition, one occurrence of technical difficulties was due to a malfunction of the microphone used for recording sound during the experiment, and one occurrence was due to difficulties in the connection between the smartphone and tablet used for the delivery of the intervention. The intervention was completed as instructed by 93.76% (15/16) of participants in the eAAMT-SP condition and 100% (13/13) of participants in the swipe control condition. Thus, the cutoff of 50% was met. Follow-up assessments were completed correctly by 68.75% (11/16) of participants in the eAAMT-SP condition and 100% (13/13) of participants in the swipe control condition. Thus, the threshold of 75% for the feasibility of the study design was not met in the eAAMT-SP condition. The feasibility evaluations were calculated with the participants who had been randomized. Regarding the usability questions, the effect size for the comparison of *intelligibility of the instructions* between the eAAMT-SP and swipe control conditions was *g*=−0.86, which constitutes a large effect. The mean rating was 4.6 (SD 0.63) in the eAAMT-SP condition and 5 (SD 0) in the swipe control condition. The between-condition effect size for *distancing from dysfunctional beliefs* was *g*=0.22, whereas the effect size for “approaching functional beliefs” was *g*=0.55, which constitutes a small and medium effect, respectively. Participants rated their ability to distance themselves from dysfunctional beliefs through the training as 3.13 (SD 0.64) in the eAAMT-SP condition and 2.85 (SD 1.82) in the swipe control condition, whereas the ability to approach functional beliefs was rated as 4.20 (SD 0.77) and 3.54 (SD 1.56), respectively. Subjective ease of displaying sadness in the eAAMT-SP condition was 1.67 (SD 1.18) before and 4.2 (SD 0.86) after the training. Subjective ease of displaying positive emotions was 3.48 (SD 1.40) before and 4.22 (SD 0.92) after the training. No substantial differences were found between the 2 conditions regarding participants’ ability to distance themselves from dysfunctional beliefs (t_26_=0.57; P=.57) or their ability to approach functional beliefs (t_26_=1.45; P=.16).

Regarding the acceptability questions, the between-condition effect size for *modification of beliefs* was *g*=0.27, which constitutes a small effect. The helpfulness of the training in modifying stress-related beliefs was rated to be 3.53 (SD 1.06) in the eAAMT-SP condition and 3.15 (SD 1.72) in the swipe control condition. The between-condition effect size for “recommendation to a friend” was *g*=0.23, which constitutes a small effect. The likelihood of recommending the training to a friend was rated as 3.60 (SD 1.40) in the eAAMT-SP condition and 3.23 (SD 1.88) in the swipe control condition. No significant between-group differences were found for “modification of beliefs*”* (t_26_=0.71; P=.48) or for the likelihood of recommending the training to a friend (t_26_=0.59; P=.56).

### Primary Clinical Outcome

**Figure 2:**
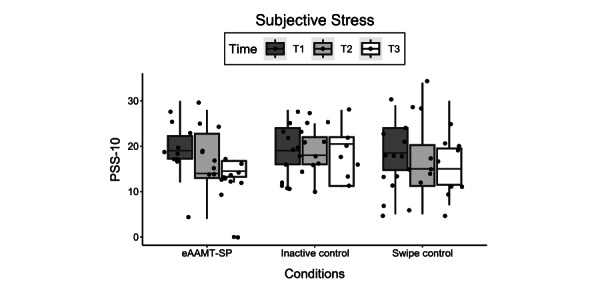
Box plots of Perceived Stress Scale (PSS) scores in the 3 conditions (emotion-based approach-avoidance modification training using sadness and positive emotions [eAAMT-SP], swipe control, and inactive control) at the preassessment (T1), postassessment (T2), and follow-up-assessment (T3) time points (N=30).

For the PSS-10, Table 2 shows that subjective stress decreased in all conditions from the pre- to postassessment time point. For pretest-posttest differences, the effect size was *g*=0.80 (95% CI −0.03 to 2.09) for the comparison between the eAAMT-SP and inactive control conditions, which constitutes a large effect. The effect size for the comparison between the eAAMT-SP and swipe control conditions was *g*=0.76 (95% CI −1.8 to 0.16) in favor of the eAAMT-SP condition, whereas the effect size for the comparison between the swipe and inactive control conditions was *g*=0.18 (95% CI −0.67 to 1.3)", which constitutes a moderate and a negligible effect, respectively. The box plot for the PSS-10 scores in the 3 conditions is shown in Figure 2.

**Table 2:** Means and SDs of primary and secondary clinical outcome variables at the preassessment time point (T1) and at the follow-up assessment (T3) for the 3 study conditions (emotion-based approach-avoidance modification training using sadness and positive emotions [eAAMT-SP], swipe control, and inactive control; N=30).

Measure	eAAMT-SP, mean (SD)		Swipe control, mean (SD)		Inactive control, mean (SD)	
	T1	T3	T1	T3	T1	T3
PSS-10^a^	20 (4.97)	13.5 (5.06)	18.7 (7.53)	16.2 (7.04)	19.6 (5.68)	18.3 (6.46)
DAS-A^b^	25.3 (7.70)	13.8 (3.64)	28.1 (12.23)	15.6 (7.62)	33 (9.07)	27 (10.30)
DAS-H^c^	17.1 (4.28)	12.2 (3.16)	16.9 (5.92)	12.7 (4.24)	21.6 (9.25)	22 (10.36)
CES-D^d^	17.7 (6.86)	10.4 (4.27)	15.6 (8.37)	11 (8.19)	21 (9.96)	18.9 (12.05)
ERSQ^e^	2.34 (0.58)	2.72 (0.49)	2.57 (0.59)	2.96 (0.51)	2.18 (0.43)	2.49 (0.41)

^a^PSS-10: 10-item Perceived Stress Scale.

^b^DAS-A: Dysfunctional Attitudes Scale form A.

^c^DAS-H: Dysfunctional Attitudes Scale form H.

^d^CES-D: Center for Epidemiologic Studies Depression Scale.

^e^ERSQ: Emotion Regulation Skills Questionnaire.

### Secondary Clinical Outcomes

Agreement with dysfunctional beliefs (DAS-A; agreement) decreased in all conditions from the pre- to postassessment time point, whereas perceived helpfulness of dysfunctional beliefs (DAS-H; perceived helpfulness) decreased in the eAAMT-SP and swipe control conditions but increased slightly in the inactive control condition (Table 2). For the DAS-A, the effect size for the pretest-posttest difference was *g*=0.79 (95% CI −0.07 to 2.1) for the comparison between the eAAMT-SP and inactive control conditions, which constitutes a moderate effect. The effect size was *g*=0.13 (95% CI −0.98 to 0.97) for the comparison between the eAAMT-SP and swipe control conditions and *g*=0.76 (95% CI −0.03 to 1.81) for the comparison between the swipe and inactive control conditions, which constitutes a negligible and a moderate effect, respectively. For the DAS-H, the effect size for the comparison of the pretest-posttest differences between the eAAMT-SP and inactive control conditions was *g*=1.02 (95% CI 0.22 to 2.24), which constitutes a large effect. The effect size for the comparison between the eAAMT-SP and swipe control conditions was *g*=−0.17 (95% CI −1.15 to 0.73) in favor of the eAAMT-SP condition, which constitutes a negligible effect. The effect size for the comparison between the swipe and inactive control conditions was *g*=0.89 (95% CI 0.03 to 1.98), which constitutes a large effect.

Table 2 shows that scores on the ERSQ increased in all 3 conditions from the pre- to postassessment time point. The effect size for the comparison of the pretest-posttest difference among the conditions was *g*=−0.14 (95% CI −1.14 to 0.74) for the comparison between the eAAMT-SP and inactive control conditions, *g*=−0.01 (95% CI −1.13 to 0.80) for the comparison between the eAAMT-SP and swipe control conditions, and *g*=−0.14 (95% CI −1.38 to 0.62) for the comparison between the swipe and inactive control conditions. Thus, the effect sizes were negligible for all 3 comparisons.

Scores on the CES-D decreased as well from the pre- to postassessment time point (Table 2). The effect size was *g*=0.55 (95% CI −0.32 to 1.55) for the comparison between the eAAMT-SP and inactive control conditions and *g*=−0.57 (95% CI −1.74 to 0.30) in favor of the eAAMT-SP condition for the comparison between the eAAMT-SP and swipe control conditions. Both effect sizes were moderate. The effect size for the comparison of pretest-posttest differences between the swipe and inactive control conditions was *g*=0.27 (95% CI −0.75 to 1.20), which constitutes a small effect.

### Exploratory ANOVAs

For the primary clinical outcome, the supplementary per-protocol ANOVA for the comparison between the eAAMT-SP and control conditions yielded a nonsignificant main effect for condition (*F*_1, 18_=1.13; P=.30) and a significant 1-tailed effect for the interaction between condition and time (*F*_1, 18_=3.49; P=.08). The follow-up comparison between the eAAMT-SP and swipe control conditions revealed a nonsignificant effect for condition (*F*_1, 18_=0.08; P=.79) and a nonsignificant interaction between time and condition (*F*_1, 18_=15.75; P=.10).

For the secondary clinical outcome *evaluation of dysfunctional beliefs*, the supplementary per-protocol ANOVA for the comparison between the eAAMT-SP and control conditions revealed a significant main effect of condition for the DAS-A (*F*_1, 18_=10.10; P=.005) and DAS-H (*F*_1, 18_=5.21; P=.04). The interaction between condition and time yielded a significant 1-tailed effect for the DAS-A (*F*_1, 18_=3.36; P=.08) and DAS-H (*F*_1, 18_=5.63; P=.03). The follow-up comparison between the eAAMT-SP and swipe control conditions did not find a significant effect of condition for the DAS-A (*F*_1, 18_=0.47; P=.50) or DAS-H (*F*_1, 18_=0.01; P=.94) or a significant interaction between time and condition for the DAS-A (*F*_1, 18_=0.09; P=.77) or DAS-H (*F*_1, 18_=0.16; P=.69). The DAS-A assessed agreement with dysfunctional beliefs, and the DAS-H assessed the perceived helpfulness of dysfunctional beliefs. For *emotion regulation skills* as assessed using the ERSQ, the supplementary per-protocol ANOVA for the comparison between the eAAMT-SP and control conditions found a nonsignificant main effect for condition (*F*_1, 18_=1.02; P=.34) and a nonsignificant interaction between condition and time (*F*_1, 18_=0.10; P=.76). For *depressive symptoms* as assessed using the CES-D, the supplementary per-protocol ANOVA for the comparison of CES-D scores in the eAAMT-SP and control conditions found a significant 1-tailed effect for condition (*F*_1, 18_=3.06; P=.10) and a nonsignificant interaction between condition and time (*F*_1, 18_=1.64; P=.22).

Missing data were imputed for 17% (5/30) of the participants. The data were missing at random. The ITT analyses did not differ significantly from the per-protocol analyses.

## Discussion

### Principal Findings

The primary goal of this pilot study was to evaluate the feasibility of a smartphone-based AAMT that uses expressions of sadness and positive emotions to move stress-enhancing beliefs away from oneself and stress-reducing beliefs toward oneself. Second, we aimed to explore the therapeutic potential of the new eAAMT-SP intervention by assessing its effects on the clinical outcomes of perceived stress, agreement with and perceived helpfulness of dysfunctional beliefs, emotion regulation, and depressive symptoms. For this purpose, we compared the eAAMT-SP condition with both an active and an inactive control condition in a sample of 30 participants with elevated stress. These findings indicate the satisfactory usability and acceptability of the novel eAAMT-SP intervention. Adherence to the intervention and feasibility of the study design were good, whereas the technical feasibility of the study design should be improved. In terms of usability and acceptability, the novel eAAMT-SP was comparable with the standard swipe AAMT condition. With regard to the effects on the PSS-10 as the primary clinical outcome, the effect sizes indicated larger reductions in subjective stress in the eAAMT-SP condition compared with either the swipe or inactive control condition. For the secondary clinical outcomes (DAS-A, DAS-H, ERSQ, and CES-D), a slightly different pattern was found, with large to moderate effects for the comparison of pretest-posttest differences between the eAAMT-SP and inactive control conditions and negligible to moderate effects for the comparison between the eAAMT-SP and swipe control conditions.

In comparison with other AAMT approaches aimed at reducing subjective stress, the eAAMT-SP used in this study appears to be more effective. Although we found a large effect for the change in subjective stress in the eAAMT-SP condition compared with the swipe and inactive control conditions, other AAMT studies targeting subjective stress found an effect exclusively in a dysphoric subgroup [48] or no effect at all [49]. There are several potential reasons for this discrepancy. First, to move stimuli in eAAMT-SP, the training requires participants not to perform the typically used swiping motion but to enact emotional expressions, which are arguably associated with a greater valence than swiping or joystick motions. Similar to the inferential mechanisms proposed by Van Dessel et al [33], the eAAMT-SP harnesses the transformative power of emotions as the greater valence of the emotional training reaction is assumed to be transferred to the stimuli, thus modifying the evaluation of these stimuli more strongly than can be expected in a training using swiping motions. Second, our training used functional and dysfunctional stress-related beliefs as training stimuli, thus targeting potentially stress-inducing belief systems directly. This is a crucial difference between our study and previous AAMT studies. Evaluating whether a belief is functional or dysfunctional may prompt reflection on the personal relevance of the stress-related beliefs and their value in one’s subjective belief system. In line with the theory of cognitive dissonance by Festinger [34], moving negatively evaluated beliefs toward oneself and positively evaluated beliefs away from oneself might create cognitive dissonance, which could be resolved by the individual through modifying the evaluations of the beliefs. Finally, we assessed subjective stress within the previous week using a self-report questionnaire, whereas Becker et al [48] assessed stress-related mood and Ferrari et al [49] assessed stress with the help of a psychophysiological index. With regard to primary clinical outcomes, the other 2 studies assessed short-term effects, whereas we assessed mid- to long-term effects. Thus, it can be speculated that the other 2 studies failed to capture a “sleeper effect” that may have contributed to the large effects found in this study. As the eAAMT-SP targets stress-related beliefs, it could be expected that it might take some time after the training until a participant’s individual belief system is modified and measurable effects emerge. However, given that some of our secondary clinical outcomes demonstrated notable mid- to long-term effects, we do not consider this a likely explanation. To identify the causes of the differences found in the 3 studies, future research needs to systematically vary the training reactions used to move the stimuli in the AAMT as well as the nature of the stimuli (pictures vs stress-relevant beliefs) while using the same instruments to assess potential effects on stress.

When compared with non-AAMT cognitive behavioral stress interventions, the effect of *g*=0.80 for subjective stress is comparable with the average effect sizes of *d*=0.65 or *d*=1.00 that have been reported in meta-analyses for cognitive behavioral stress management interventions [19,20]. Thus, although the effect sizes of this pilot study should be interpreted with great caution (as the study was not sufficiently powered to allow for confirmatory testing), the findings of this study provide preliminary evidence that the novel eAAMT-SP intervention seems to be comparable with the gold standard of treatment for subjective stress (although the AAMT treatment was notably shorter than the cognitive behavioral interventions included in the meta-analyses).

With regard to the effects on the secondary clinical outcome variables, it is of note that the effect size of *g*=0.55 for the reduction in depressive symptoms in the eAAMT-SP condition compared with the inactive control condition is slightly smaller than the effect reported by Lukas et al [37], who found an effect of *d*=1.41 for the reduction in depressive symptoms. These differences can be assumed to result from the fact that Lukas et al [37] specifically targeted depression and, hence, used stimuli specifically developed for this particular disorder, whereas this study focused exclusively on stress, which is associated with but not identical to depression. Regarding emotion regulation skills, the effect of *g*=−0.14 in the eAAMT-SP condition compared with the inactive control condition was also lower than that of the study by Lukas et al [94], who found an effect of *d*=0.97. Although a smaller effect on emotion regulation could be expected as the training developed by Lukas et al [94] focused on improving emotion recognition, the absence of any positive effect came as a surprise. It could be expected that purposefully expressing different emotions to move stimuli in eAAMT-SP would improve participants’ emotion regulation skills. This unexpected finding can potentially be explained by the exclusive focus on sadness as a typically undesired emotion. It can be hypothesized that eAAMTs that use more than one undesired affective state would facilitate participants’ ability to identify, understand, accept, and modify a broad range of undesired affective states. In addition, Lukas et al [94] only assessed one facet of emotion regulation (emotion recognition), whereas we aimed to assess emotion regulation more broadly. Moreover, previous studies investigating the effects of AAMT on emotion regulation combined AAMT with face-to-face psychoeducation, which is likely to further enhance the clinical efficacy of the intervention [43,44].

This study has several limitations. First, as an exploratory pilot trial, this study had a small sample size and, therefore, was not powered to perform confirmatory testing. Hence, statistically significant differences between the study conditions were not expected, and small effects could not be detected. A larger trial will be needed to robustly assess the clinical efficacy of our intervention. Second, this training was only performed for 20 to 30 minutes on 4 consecutive days, whereas in other studies targeting clinical outcomes, participants performed AAMTs for a period of approximately 14 days [37,43,44]. Thus, the effects of this study may underestimate those resulting from a more sustained use of the intervention. Similarly, the follow-up period of 1 week was very short and may have failed to capture the long-term effects of the intervention. Third, this study differed from various previous studies with regard to both using the display of emotions to move stimuli (instead of joystick or swiping movements) and using beliefs as stimuli (instead of pictures of real objects). Both features can be considered promising deviations from previous AAMTs as emotions carry stronger valence than simple wrist or finger movements. Furthermore, swiping away stress-enhancing beliefs can be argued to be more effective than swiping away pictures of objects cueing stress responses as the latter likely leads to a more negative evaluation of these objects and, hence, a stronger stress response whenever these objects are encountered. However, the simultaneous implementation of both of these innovations interferes with clarifying which of them is responsible for effect sizes greater than those found in previous studies. Fourth, when exploring the therapeutic potential of the intervention, we exclusively used self-report measures. Fifth, the sample consisted largely of university students and is, therefore, not representative of the general population. Sixth, recruitment took place over a period of 2 years, which was due to slow recruitment during the COVID-19 pandemic. Although all 3 groups were recruited over the entire recruitment period, which should control for random effects on perceived stress, cohort effects may be present. Finally, we did not directly compare the intervention with the current gold standard of psychological interventions for elevated stress.

Thus, future research should work to replicate these findings in a randomized clinical trial sufficiently powered to allow for confirmatory testing. Ideally, the sample should be representative of the population of individuals with elevated stress. Moreover, the study should include a comparison with a gold-standard intervention for the reduction of stress. Future studies should also systematically compare the efficacy of all possible combinations of (1) using emotions versus finger movements to move stimuli and (2) using stress-related beliefs versus pictures of specific objects cueing stress. Furthermore, in future studies, outcomes should include not only self-report measures but also biological and psychophysiological indicators of stress as well as an assessment of approach-avoidance biases toward stress-related beliefs. To assess the long-term effects of the intervention, the period for the follow-up assessment should be extended to several weeks or even months. Finally, it is of note that specific dysfunctional beliefs that maintain a clinical problem can be identified not only for elevated stress but also for psychiatric disorders such as depression, eating disorders, alcohol use disorders, and anxiety disorders [95-99]. Thus, future studies should evaluate the efficacy of disorder-specific versions of eAAMT-SPs as an (adjunctive) treatment for these disorders.

### Conclusions

This randomized controlled pilot study explored the feasibility and clinical efficacy of an eAAMT-SP using expressions of sadness versus positive emotions for reducing subjective stress. The findings of this study indicate good feasibility and acceptability of the intervention and an advantage of the eAAMT-SP over the standard swipe AAMT or an inactive control condition, with need for improvement regarding the technical feasibility of the design. The effect size for the primary clinical outcome indicates a potential advantage of the eAAMT-SP over the standard swipe AAMT with regard to the reduction of perceived stress. This suggests that incorporating emotions into the AAMT paradigm may have significant therapeutic potential. This should be explored further in fully powered randomized controlled trials. As dysfunctional beliefs and emotion regulation difficulties are implicated in the etiology and maintenance of various other mental disorders, future studies should evaluate the efficacy of disorder-specific eAAMT-SP versions—either as stand-alone interventions or as add-on components in evidence-based treatments for these disorders.

## Appendix

Multimedia Appendix 1 CONSORT-eHEALTH checklist (V 1.6.1)

## Abbreviations

AAMT: approach-avoidance modification training
CES-D: Center for Epidemiologic Studies Depression Scale
DAS: Dysfunctional Attitudes Scale
DAS-A: Dysfunctional Attitudes Scale form A
DAS-H: Dysfunctional Attitudes Scale form H
eAAMT: emotion-based approach-avoidance modification training
eAAMT-SP: emotion-based approach-avoidance modification training using sadness and positive emotions
ERSQ: Emotion Regulation Skills Questionnaire
ITT: intention to treat
PSS-10: 10-item Perceived Stress Scale

## Data Availability

The anonymized data sets and codes used for the statistical evaluations are publicly available on the Open Science Framework platform [100].
