# Supplementary material for: Sadness-Based Approach-Avoidance Modification Training for Subjective Stress in Adults: Pilot Randomized Controlled Trial
Source: JMIR Form Res. 2023 Nov 30;7:e50324. doi: 10.2196/50324 (PMC10722377; doi:10.2196/50324)
Supplement: Multimedia Appendix 1 [file formative_v7i1e50324_app1.pdf]

CONSORT-EHEALTH (V 1.6.1) - Submission/Publication Form

The CONSORT-EHEALTH checklist is intended for authors of randomized trials evaluating web-based and Internet-based applications/interventions, including mobile interventions, electronic games (incl multiplayer games), social media, certain telehealth applications, and other interactive and/or networked electronic applications. Some of the items (e.g. all subitems under item 5 - description of the intervention) may also be applicable for other study designs.

The goal of the CONSORT EHEALTH checklist and guideline is to be
a) a guide for reporting for authors of RCTs,
b) to form a basis for appraisal of an ehealth trial (in terms of validity)

CONSORT-EHEALTH items/subitems are MANDATORY reporting items for studies published in the Journal of Medical Internet Research and other journals / scientific societies endorsing the checklist.

Items numbered 1., 2., 3., 4a., 4b etc are original CONSORT or CONSORT-NPT (non-pharmacologic treatment) items.
Items with Roman numerals (i., ii, iii, iv etc.) are CONSORT-EHEALTH extensions/clarifications.

As the CONSORT-EHEALTH checklist is still considered in a formative stage, we would ask that you also RATE ON A SCALE OF 1-5 how important/useful you feel each item is FOR THE PURPOSE OF THE CHECKLIST and reporting guideline (optional).

Mandatory reporting items are marked with a red *.
In the textboxes, either copy & paste the relevant sections from your manuscript into this form - please include any quotes from your manuscript in QUOTATION MARKS,
or answer directly by providing additional information not in the manuscript, or elaborating on why the item was not relevant for this study.

YOUR ANSWERS WILL BE PUBLISHED AS A SUPPLEMENTARY FILE TO YOUR PUBLICATION IN JMIR AND ARE CONSIDERED PART OF YOUR PUBLICATION (IF ACCEPTED).
Please fill in these questions diligently. Information will not be copyedited, so please use proper spelling and grammar, use correct capitalization, and avoid abbreviations.

DO NOT FORGET TO SAVE AS PDF _AND_ CLICK THE SUBMIT BUTTON SO YOUR ANSWERS ARE IN OUR DATABASE !!!

Citation Suggestion (if you append the pdf as Appendix we suggest to cite this paper in the caption):
[illegible]

Ihre Antwort ist zu lang. Kürzen Sie einige Sätze in Ihrer Antwort.

[illegible]

J Med Internet Res 2011;13(4):e126
URL: http://www.jmir.org/2011/4/e126/
doi: 10.2196/jmir.1923
PMID: 22209829

ly.rupp@googlemail.com Konto wechseln

Nicht freigegeben

Entwurf gespeichert

* Gibt eine erforderliche Frage an

Your name *

First Last

Lydia Helene Rupp

Primary Affiliation (short), City, Country *

University of Toronto, Toronto, Canada

Friedrich-Alexander-Universität Erlangen-Nürn

Your e-mail address *

abc@gmail.com

lydia.rupp@fau.de

Title of your manuscript *

Provide the (draft) title of your manuscript.

Sadness-Based Approach-Avoidance Modification Training for Subjective Stress in Adults: Pilot Randomized- Controlled Trial

Ihre Antwort ist zu lang. Kürzen Sie einige Sätze in Ihrer Antwort.

Name of your App/Software/Intervention *

If there is a short and a long/alternate name, write the short name first and add the long name in brackets.

WIZ-Stress

Evaluated Version (if any)

e.g. "V1", "Release 2017-03-01", "Version 2.0.27913"

Not applicable

Language(s) *

What language is the intervention/app in? If multiple languages are available, separate by comma (e.g. "English, French")

German

URL of your Intervention Website or App

e.g. a direct link to the mobile app on app in appstore (itunes, Google Play), or URL of the website. If the intervention is a DVD or hardware, you can also link to an Amazon page.

Meine Antwort

URL of an image/screenshot (optional)

Meine Antwort

Ihre Antwort ist zu lang. Kürzen Sie einige Sätze in Ihrer Antwort.

Accessibility *

Can an enduser access the intervention presently?

- ○ access is free and open
- ○ access only for special usergroups, not open
- ○ access is open to everyone, but requires payment/subscription/in-app purchases
- ○ app/intervention no longer accessible
- ● Sonstiges: This application has been developed for research purposes and

Primary Medical Indication/Disease/Condition *

e.g. "Stress", "Diabetes", or define the target group in brackets after the condition, e.g. "Autism (Parents of children with)", "Alzheimers (Informal Caregivers of)"

Stress

Primary Outcomes measured in trial *

comma-separated list of primary outcomes reported in the trial

Perceived Stress (assessed with the PSS-10 (

Secondary/other outcomes

Are there any other outcomes the intervention is expected to affect?

Dysfunctional attitudes (assessed with the DAS (Rojas et al., 2015)), emotion regulation (assessed with the ERSQ (Berking & Znoj, 2008)), and depressive symptoms (assessed with the German version of the CES-D (Hautzinger et al., 2012))

Ihre Antwort ist zu lang. Kürzen Sie einige Sätze in Ihrer Antwort.

Recommended "Dose" *

What do the instructions for users say on how often the app should be used?

- ○ Approximately Daily
- ○ Approximately Weekly
- ○ Approximately Monthly
- ○ Approximately Yearly
- ◉ "as needed"
- ○ Sonstiges:

Approx. Percentage of Users (starters) still using the app as recommended after 3 months *

- ◉ unknown / not evaluated
- ○ 0-10%
- ○ 11-20%
- ○ 21-30%
- ○ 31-40%
- ○ 41-50%
- ○ 51-60%
- ○ 61-70%
- ○ 71%-80%
- ○ 81-90%
- ○ 91-100%
- ○ Sonstiges:

Ihre Antwort ist zu lang. Kürzen Sie einige Sätze in Ihrer Antwort.

Overall, was the app/intervention effective? *

- ○ yes: all primary outcomes were significantly better in intervention group vs control
- ◉ partly: SOME primary outcomes were significantly better in intervention group vs control
- ○ no statistically significant difference between control and intervention
- ○ potentially harmful: control was significantly better than intervention in one or more outcomes
- ○ inconclusive: more research is needed
- ○ Sonstiges:

Article Preparation Status/Stage *

At which stage in your article preparation are you currently (at the time you fill in this form)

- ○ not submitted yet - in early draft status
- ○ not submitted yet - in late draft status, just before submission
- ○ submitted to a journal but not reviewed yet
- ○ submitted to a journal and after receiving initial reviewer comments
- ◉ submitted to a journal and accepted, but not published yet
- ○ published
- ○ Sonstiges:

Ihre Antwort ist zu lang. Kürzen Sie einige Sätze in Ihrer Antwort.

Journal *

If you already know where you will submit this paper (or if it is already submitted), please provide the journal name (if it is not JMIR, provide the journal name under "other")

- ( ) not submitted yet / unclear where I will submit this
- ( ) Journal of Medical Internet Research (JMIR)
- ( ) JMIR mHealth and UHealth
- ( ) JMIR Serious Games
- ( ) JMIR Mental Health
- ( ) JMIR Public Health
- (•) JMIR Formative Research
- ( ) Other JMIR sister journal
- ( ) Sonstiges:

Is this a full powered effectiveness trial or a pilot/feasibility trial? *

- (•) Pilot/feasibility
- ( ) Fully powered

Manuscript tracking number *

If this is a JMIR submission, please provide the manuscript tracking number under "other" (The ms tracking number can be found in the submission acknowledgement email, or when you login as author in JMIR. If the paper is already published in JMIR, then the ms tracking number is the four-digit number at the end of the DOI, to be found at the bottom of each published article in JMIR)

- ( ) no ms number (yet) / not (yet) submitted to / published in JMIR
- (•) Sonstiges: #50324

Ihre Antwort ist zu lang. Kürzen Sie einige Sätze in Ihrer Antwort.

TITLE AND ABSTRACT

1a) TITLE: Identification as a randomized trial in the title

1a) Does your paper address CONSORT item 1a? *

I.e does the title contain the phrase "Randomized Controlled Trial"? (if not, explain the reason under "other")

- (x) yes
- ( ) Sonstiges:

1a-i) Identify the mode of delivery in the title

Identify the mode of delivery. Preferably use “web-based” and/or “mobile” and/or “electronic game” in the title. Avoid ambiguous terms like “online”, “virtual”, “interactive”. Use “Internet-based” only if Intervention includes non-web-based Internet components (e.g. email), use “computer-based” or “electronic” only if offline products are used. Use “virtual” only in the context of “virtual reality” (3-D worlds). Use “online” only in the context of “online support groups”. Complement or substitute product names with broader terms for the class of products (such as “mobile” or “smart phone” instead of “iphone”), especially if the application runs on different platforms.

	1	2	3	4	5	
subitem not at all important	( )	( )	(x)	( )	( )	essential

Auswahl löschen

Ihre Antwort ist zu lang. Kürzen Sie einige Sätze in Ihrer Antwort.

Does your paper address subitem 1a-i? *

Copy and paste relevant sections from manuscript title (include quotes in quotation marks "like this" to indicate direct quotes from your manuscript), or elaborate on this item by providing additional information not in the ms, or briefly explain why the item is not applicable/relevant for your study

This study focuses on evaluating the use of emotions in an approach-avoidance modification training, thus we chose to focus on this novel aspect in the title.

1a-ii) Non-web-based components or important co-interventions in title

Mention non-web-based components or important co-interventions in title, if any (e.g., “with telephone support”).

	1	2	3	4	5	
subitem not at all important	○	○	○	◉	○	essential

Auswahl löschen

Does your paper address subitem 1a-ii?

Copy and paste relevant sections from manuscript title (include quotes in quotation marks "like this" to indicate direct quotes from your manuscript), or elaborate on this item by providing additional information not in the ms, or briefly explain why the item is not applicable/relevant for your study

This study does not include any non-web-based intervention components.

Ihre Antwort ist zu lang. Kürzen Sie einige Sätze in Ihrer Antwort.

1a-iii) Primary condition or target group in the title

Mention primary condition or target group in the title, if any (e.g., “for children with Type I Diabetes”) Example: A Web-based and Mobile Intervention with Telephone Support for Children with Type I Diabetes: Randomized Controlled Trial

	1	2	3	4	5	
subitem not at all important	○	○	○	○	◉	essential

Auswahl löschen

Does your paper address subitem 1a-iii? *

Copy and paste relevant sections from manuscript title (include quotes in quotation marks "like this" to indicate direct quotes from your manuscript), or elaborate on this item by providing additional information not in the ms, or briefly explain why the item is not applicable/relevant for your study

"Sadness-Based Approach-Avoidance Modification Training for Subjective Stress in Adults: Pilot Randomized- Controlled Pilot Trial"

1b) ABSTRACT: Structured summary of trial design, methods, results, and conclusions

NPT extension: Description of experimental treatment, comparator, care providers, centers, and blinding status.

Ihre Antwort ist zu lang. Kürzen Sie einige Sätze in Ihrer Antwort.

1b-i) Key features/functionalities/components of the intervention and comparator in the METHODS section of the ABSTRACT

Mention key features/functionalities/components of the intervention and comparator in the abstract. If possible, also mention theories and principles used for designing the site. Keep in mind the needs of systematic reviewers and indexers by including important synonyms. (Note: Only report in the abstract what the main paper is reporting. If this information is missing from the main body of text, consider adding it)

	1	2	3	4	5	
subitem not at all important	○	○	○	○	◉	essential

Auswahl löschen

Does your paper address subitem 1b-i? *

Copy and paste relevant sections from the manuscript abstract (include quotes in quotation marks "like this" to indicate direct quotes from your manuscript), or elaborate on this item by providing additional information not in the ms, or briefly explain why the item is not applicable/relevant for your study

"We aimed to evaluate the feasibility of a mobile emotion-enhanced AAMT that engages users to display sadness to move stress-enhancing beliefs away and display positive emotions to move stress-reducing beliefs towards themselves (emotion-based AAMT using sadness and positive emotions [eAAMT-SP]). Additionally, we explored the clinical efficacy of this novel intervention." and "We allocated N = 30 adult individuals with elevated stress randomly to 1 of 3 conditions (eAAMT-SP, a swipe control condition, and an inactive control condition)"

Ihre Antwort ist zu lang. Kürzen Sie einige Sätze in Ihrer Antwort.

1b-ii) Level of human involvement in the METHODS section of the ABSTRACT

Clarify the level of human involvement in the abstract, e.g., use phrases like “fully automated” vs. “therapist/nurse/care provider/physician-assisted” (mention number and expertise of providers involved, if any). (Note: Only report in the abstract what the main paper is reporting. If this information is missing from the main body of text, consider adding it)

	1	2	3	4	5	
subitem not at all important	○	○	◉	○	○	essential

Auswahl löschen

Does your paper address subitem 1b-ii?

Copy and paste relevant sections from the manuscript abstract (include quotes in quotation marks "like this" to indicate direct quotes from your manuscript), or elaborate on this item by providing additional information not in the ms, or briefly explain why the item is not applicable/relevant for your study

We have not included this information in the abstract, as this study was conducted in a laboratory setting to assess the feasibility of the intervention.

Ihre Antwort ist zu lang. Kürzen Sie einige Sätze in Ihrer Antwort.

1b-iii) Open vs. closed, web-based (self-assessment) vs. face-to-face assessments in the METHODS section of the ABSTRACT

Mention how participants were recruited (online vs. offline), e.g., from an open access website or from a clinic or a closed online user group (closed usergroup trial), and clarify if this was a purely web-based trial, or there were face-to-face components (as part of the intervention or for assessment). Clearly say if outcomes were self-assessed through questionnaires (as common in web-based trials). Note: In traditional offline trials, an open trial (open-label trial) is a type of clinical trial in which both the researchers and participants know which treatment is being administered. To avoid confusion, use “blinded” or “unblinded” to indicated the level of blinding instead of “open”, as “open” in web-based trials usually refers to “open access” (i.e. participants can self-enrol). (Note: Only report in the abstract what the main paper is reporting. If this information is missing from the main body of text, consider adding it)

	1	2	3	4	5	
subitem not at all important	○	○	◉	○	○	essential

Auswahl löschen

Does your paper address subitem 1b-iii?

Copy and paste relevant sections from the manuscript abstract (include quotes in quotation marks "like this" to indicate direct quotes from your manuscript), or elaborate on this item by providing additional information not in the ms, or briefly explain why the item is not applicable/relevant for your study

"To explore the clinical efficacy of the intervention, we compared pretest-posttest differences in perceived stress (primary clinical outcome) and 3 secondary clinical outcomes (agreement with and perceived helpfulness of dysfunctional beliefs, emotion regulation, and depressive symptoms) among the conditions." Outcome assessment took place in the laboratory for two of the groups and at home for the inactive control group.

Ihre Antwort ist zu lang. Kürzen Sie einige Sätze in Ihrer Antwort.

1b-iv) RESULTS section in abstract must contain use data

Report number of participants enrolled/assessed in each group, the use/uptake of the intervention (e.g., attrition/adherence metrics, use over time, number of logins etc.), in addition to primary/secondary outcomes. (Note: Only report in the abstract what the main paper is reporting. If this information is missing from the main body of text, consider adding it)

	1	2	3	4	5	
subitem not at all important	○	○	◉	○	○	essential

Auswahl löschen

Does your paper address subitem 1b-iv?

Copy and paste relevant sections from the manuscript abstract (include quotes in quotation marks "like this" to indicate direct quotes from your manuscript), or elaborate on this item by providing additional information not in the ms, or briefly explain why the item is not applicable/relevant for your study

"We allocated N = 30 adult individuals with elevated stress randomly to 1 of 3 conditions (eAAMT-SP, a swipe control condition, and an inactive control condition). We evaluated the feasibility of the intervention (technical problems, adherence, usability, and acceptability of the intervention)." We did not collect use data, as the application was only used by participants while they participated in the study in the laboratory.

1b-v) CONCLUSIONS/DISCUSSION in abstract for negative trials

Conclusions/Discussions in abstract for negative trials: Discuss the primary outcome - if the trial is negative (primary outcome not changed), and the intervention was not used, discuss whether negative results are attributable to lack of uptake and discuss reasons. (Note: Only report in the abstract what the main paper is reporting. If this information is missing from the main body of text, consider adding it)

	1	2	3	4	5	
subitem not at all important	○	○	○	◉	○	essential

Auswahl löschen

Ihre Antwort ist zu lang. Kürzen Sie einige Sätze in Ihrer Antwort.

Does your paper address subitem 1b-v?

Copy and paste relevant sections from the manuscript abstract (include quotes in quotation marks "like this" to indicate direct quotes from your manuscript), or elaborate on this item by providing additional information not in the ms, or briefly explain why the item is not applicable/relevant for your study

This does not apply, as we did not conduct a negative trial.

INTRODUCTION

2a) In INTRODUCTION: Scientific background and explanation of rationale

2a-i) Problem and the type of system/solution

Describe the problem and the type of system/solution that is object of the study: intended as stand-alone intervention vs. incorporated in broader health care program? Intended for a particular patient population? Goals of the intervention, e.g., being more cost-effective to other interventions, replace or complement other solutions? (Note: Details about the intervention are provided in “Methods” under 5)

	1	2	3	4	5	
subitem not at all important	○	○	○	○	◉	essential

Auswahl löschen

Ihre Antwort ist zu lang. Kürzen Sie einige Sätze in Ihrer Antwort.

Does your paper address subitem 2a-i? *

Copy and paste relevant sections from the manuscript (include quotes in quotation marks "like this" to indicate direct quotes from your manuscript), or elaborate on this item by providing additional information not in the ms, or briefly explain why the item is not applicable/relevant for your study

"As chronic stress affects up to 33% of the adult population [6], [,7], there is a significant need for low-threshold and accessible interventions for effective stress management." "Building on these studies, we propose that AAMTs targeting subjective stress might be more effective if they explicitly address stress-related beliefs (instead of pictures of positive or negative stimuli)."

2a-ii) Scientific background, rationale: What is known about the (type of) system

Scientific background, rationale: What is known about the (type of) system that is the object of the study (be sure to discuss the use of similar systems for other conditions/diagnoses, if appropiate), motivation for the study, i.e. what are the reasons for and what is the context for this specific study, from which stakeholder viewpoint is the study performed, potential impact of findings [2]. Briefly justify the choice of the comparator.

	1	2	3	4	5	
subitem not at all important	○	●	○	○	○	essential

Auswahl löschen

Does your paper address subitem 2a-ii? *

Copy and paste relevant sections from the manuscript (include quotes in quotation marks "like this" to indicate direct quotes from your manuscript), or elaborate on this item by providing additional information not in the ms, or briefly explain why the item is not applicable/relevant for your study

"efficacy of AAMTs has been demonstrated in various clinical applications. Significant effects on clinical outcomes have been found for alcohol use disorder [35], [,36], depressive symptoms [37], [,38], social anxiety disorder [39], [,40], cannabis use [41], body dissatisfaction and other symptoms of eating disorders [42], [,43], as well as in procrastination [44]." We used a standard swipe AAMT as the comparator (for further information see manuscript, heading "Background")

Ihre Antwort ist zu lang. Kürzen Sie einige Sätze in Ihrer Antwort.

2b) In INTRODUCTION: Specific objectives or hypotheses

Does your paper address CONSORT subitem 2b? *

Copy and paste relevant sections from the manuscript (include quotes in quotation marks "like this" to indicate direct quotes from your manuscript), or elaborate on this item by providing additional information not in the ms, or briefly explain why the item is not applicable/relevant for your study

"Thus, the primary goal of this study was to explore the feasibility of an innovative smartphone-based eAAMT that uses expressions of sadness to foster avoidance of stress-enhancing beliefs and expressions of positive emotions to foster an approach towards stress-reducing beliefs."

METHODS

3a) Description of trial design (such as parallel, factorial) including allocation ratio

Does your paper address CONSORT subitem 3a? *

Copy and paste relevant sections from the manuscript (include quotes in quotation marks "like this" to indicate direct quotes from your manuscript), or elaborate on this item by providing additional information not in the ms, or briefly explain why the item is not applicable/relevant for your study

"We are reporting the results of an 8-armed randomized controlled trial that aimed to evaluate the feasibility and explore the clinical efficacy of several variations of the new eAAMT paradigm in the context of elevated stress (N = 82)."

3b) Important changes to methods after trial commencement (such as eligibility criteria), with reasons

Ihre Antwort ist zu lang. Kürzen Sie einige Sätze in Ihrer Antwort.

Does your paper address CONSORT subitem 3b? *

Copy and paste relevant sections from the manuscript (include quotes in quotation marks "like this" to indicate direct quotes from your manuscript), or elaborate on this item by providing additional information not in the ms, or briefly explain why the item is not applicable/relevant for your study

No major changes to the study procedure were made after trial commencement. Only participant compensation was adapted. (for further information see manuscript, heading "Participants and Procedure")

3b-i) Bug fixes, Downtimes, Content Changes

Bug fixes, Downtimes, Content Changes: ehealth systems are often dynamic systems. A description of changes to methods therefore also includes important changes made on the intervention or comparator during the trial (e.g., major bug fixes or changes in the functionality or content) (5-iii) and other “unexpected events” that may have influenced study design such as staff changes, system failures/downtimes, etc. [2].

	1	2	3	4	5	
subitem not at all important	○	○	◉	○	○	essential

Auswahl löschen

Does your paper address subitem 3b-i?

Copy and paste relevant sections from the manuscript (include quotes in quotation marks "like this" to indicate direct quotes from your manuscript), or elaborate on this item by providing additional information not in the ms, or briefly explain why the item is not applicable/relevant for your study

This item does not apply, as the application did not undergo any major changes during the trial.

4a) Eligibility criteria for participants

Ihre Antwort ist zu lang. Kürzen Sie einige Sätze in Ihrer Antwort.

Does your paper address CONSORT subitem 4a? *

Copy and paste relevant sections from the manuscript (include quotes in quotation marks "like this" to indicate direct quotes from your manuscript), or elaborate on this item by providing additional information not in the ms, or briefly explain why the item is not applicable/relevant for your study

"Participants were included in the study if they (1) reported elevated perceived stress as indicated by a score of ≥19 or higher on the 10-item- version of the Perceived Stress Scale (PSS-10) [67], (2) were aged ≥18 years or older, and (3) provided informed consent for study participation. Exclusion criteria were (1) the presence of psychotic disorders, (2) physical impairments of emotion expression (e.g., facial paralysis), (3) heavy smoking (i.e., > 10 cigarettes per day; owing to the parallel collection of cortisol samples, it is not reported in this paper due to a deviating focus of this manuscript [65], [,68]), and (4) current suicidal ideation. A cut-off score of 19 on the PSS-10 was defined as 1 SD above the mean score in the German version of the PSS-10 [67]. This was implemented according to the suggested procedures by Heber et al [69]."

4a-i) Computer / Internet literacy

Computer / Internet literacy is often an implicit “de facto” eligibility criterion - this should be explicitly clarified.

	1	2	3	4	5	
subitem not at all important	○	○	○	◉	○	essential

Auswahl löschen

Does your paper address subitem 4a-i?

Copy and paste relevant sections from the manuscript (include quotes in quotation marks "like this" to indicate direct quotes from your manuscript), or elaborate on this item by providing additional information not in the ms, or briefly explain why the item is not applicable/relevant for your study

We did not assess this as an inclusion or exclusion criterion. Participants were supervised by a research assistant during the entire time of their interaction with the application and were able to ask questions at any time. Thus, we expect that participants with lower computer literacy were also able to interact with the application as intended.

Ihre Antwort ist zu lang. Kürzen Sie einige Sätze in Ihrer Antwort.

4a-ii) Open vs. closed, web-based vs. face-to-face assessments:

Open vs. closed, web-based vs. face-to-face assessments: Mention how participants were recruited (online vs. offline), e.g., from an open access website or from a clinic, and clarify if this was a purely web-based trial, or there were face-to-face components (as part of the intervention or for assessment), i.e., to what degree got the study team to know the participant. In online-only trials, clarify if participants were quasi-anonymous and whether having multiple identities was possible or whether technical or logistical measures (e.g., cookies, email confirmation, phone calls) were used to detect/prevent these.

	1	2	3	4	5	
subitem not at all important	○	○	○	○	◉	essential

Auswahl löschen

Does your paper address subitem 4a-ii? *

Copy and paste relevant sections from the manuscript (include quotes in quotation marks "like this" to indicate direct quotes from your manuscript), or elaborate on this item by providing additional information not in the ms, or briefly explain why the item is not applicable/relevant for your study

"Recruiting took place in Erlangen, Germany, via posts on social networks, email newsletters of the Friedrich-Alexander-Universität Erlangen-Nürnberg, and flyers posted in public places between July 2020 and May 2022. Psychology students received course credit in compensation for their participation. In- and exclusion criteria were assessed with the help of the web-based questionnaire tool Unipark [66]."

4a-iii) Information giving during recruitment

Information given during recruitment. Specify how participants were briefed for recruitment and in the informed consent procedures (e.g., publish the informed consent documentation as appendix, see also item X26), as this information may have an effect on user self-selection, user expectation and may also bias results.

	1	2	3	4	5	
subitem not at all important	○	○	○	◉	○	essential

Ihre Antwort ist zu lang. Kürzen Sie einige Sätze in Ihrer Antwort.

Does your paper address subitem 4a-iii?

Copy and paste relevant sections from the manuscript (include quotes in quotation marks "like this" to indicate direct quotes from your manuscript), or elaborate on this item by providing additional information not in the ms, or briefly explain why the item is not applicable/relevant for your study

Participants were recruited via flyers. Interested participants could enter a link to a web-based screening tool, which included a consent form explaining the procedure of the

4b) Settings and locations where the data were collected

Does your paper address CONSORT subitem 4b? *

Copy and paste relevant sections from the manuscript (include quotes in quotation marks "like this" to indicate direct quotes from your manuscript), or elaborate on this item by providing additional information not in the ms, or briefly explain why the item is not applicable/relevant for your study

The study was conducted in a laboratory setting. Participants attended the training sessions in person and were supervised by a research assistant. "In the eAAMT-SP and the swipe control conditions, participants completed a smartphone-based training on 4 consecutive days. The training was conducted by research assistants (8 bachelor's students, 4 master's students, and 1 doctoral candidate) in psychology who were trained and supervised by a clinical psychologist with a master's degree in psychology."
"Participants in the inactive control condition received the outcome questionnaires via e-mail without participating in any kind of intervention."

4b-i) Report if outcomes were (self-)assessed through online questionnaires

Clearly report if outcomes were (self-)assessed through online questionnaires (as common in web-based trials) or otherwise.

	1	2	3	4	5	
subitem not at all important	○	○	○	○	◉	essential

Auswahl löschen

Ihre Antwort ist zu lang. Kürzen Sie einige Sätze in Ihrer Antwort.

Does your paper address subitem 4b-i? *

Copy and paste relevant sections from the manuscript (include quotes in quotation marks "like this" to indicate direct quotes from your manuscript), or elaborate on this item by providing additional information not in the ms, or briefly explain why the item is not applicable/relevant for your study

While study participation took place in person, the questionnaires were presented with a web-based tool for the eAAMT-SP and swipe control groups. The inactive control group completed the questionnaires at home, also using the web-based tool. "Outcome measures were assessed at 3 time points. The first assessment (pre-assessment time point; T1) was conducted immediately before the first training session, the second assessment (T2) was conducted after the last (i.e., fourth) training session, and the third assessment (post-assessment time point; T3) was conducted 1 week after T2. For all assessments, questionnaires were administered via the web-based survey tool Unipark

4b-ii) Report how institutional affiliations are displayed

Report how institutional affiliations are displayed to potential participants [on ehealth media], as affiliations with prestigious hospitals or universities may affect volunteer rates, use, and reactions with regards to an intervention.(Not a required item – describe only if this may bias results)

	1	2	3	4	5	
subitem not at all important	○	○	○	○	◉	essential

Auswahl löschen

Does your paper address subitem 4b-ii?

Copy and paste relevant sections from the manuscript (include quotes in quotation marks "like this" to indicate direct quotes from your manuscript), or elaborate on this item by providing additional information not in the ms, or briefly explain why the item is not applicable/relevant for your study

The application was developed for research purposes within the university and no external affiliations were present.

Ihre Antwort ist zu lang. Kürzen Sie einige Sätze in Ihrer Antwort.

5) The interventions for each group with sufficient details to allow replication, including how and when they were actually administered

5-i) Mention names, credential, affiliations of the developers, sponsors, and owners

Mention names, credential, affiliations of the developers, sponsors, and owners [6] (if authors/evaluators are owners or developer of the software, this needs to be declared in a "Conflict of interest" section or mentioned elsewhere in the manuscript).

	1	2	3	4	5	
subitem not at all important	○	○	○	○	◉	essential

Auswahl löschen

Does your paper address subitem 5-i?

Copy and paste relevant sections from the manuscript (include quotes in quotation marks "like this" to indicate direct quotes from your manuscript), or elaborate on this item by providing additional information not in the ms, or briefly explain why the item is not applicable/relevant for your study

This does not apply to the present study, as the application was not developed by a company on the free market. The application was designed by a PhD candidate as a part of this research project.

5-ii) Describe the history/development process

Describe the history/development process of the application and previous formative evaluations (e.g., focus groups, usability testing), as these will have an impact on adoption/use rates and help with interpreting results.

	1	2	3	4	5	
subitem not at all important	○	○	◉	○	○	essential

Auswahl löschen

Ihre Antwort ist zu lang. Kürzen Sie einige Sätze in Ihrer Antwort.

Does your paper address subitem 5-ii?

Copy and paste relevant sections from the manuscript (include quotes in quotation marks "like this" to indicate direct quotes from your manuscript), or elaborate on this item by providing additional information not in the ms, or briefly explain why the item is not applicable/relevant for your study

This is the first version of the application that was developed. Our aim for this study was to evaluate the feasibility of the application and use this during further development.

5-iii) Revisions and updating

Revisions and updating. Clearly mention the date and/or version number of the application/intervention (and comparator, if applicable) evaluated, or describe whether the intervention underwent major changes during the evaluation process, or whether the development and/or content was “frozen” during the trial. Describe dynamic components such as news feeds or changing content which may have an impact on the replicability of the intervention (for unexpected events see item 3b).

	1	2	3	4	5	
subitem not at all important	○	○	◉	○	○	essential

Auswahl löschen

Does your paper address subitem 5-iii?

Copy and paste relevant sections from the manuscript (include quotes in quotation marks "like this" to indicate direct quotes from your manuscript), or elaborate on this item by providing additional information not in the ms, or briefly explain why the item is not applicable/relevant for your study

This does not apply to the current study, as the content used in the application was not dynamic. Each participant received the same version of the application. The application was developed for research purposes and only had the functionality for the training paradigm that was evaluated in this study. Only the stimuli were randomly selected for each participant: "For each training session, 60 beliefs were randomly selected from a pool of 78 beliefs (30 stress-enhancing beliefs and 30 stress-reducing beliefs, randomly selected from a pool of 48 beliefs) with a 50:50 ratio for the presentation of stress-reducing and stress-enhancing beliefs."

Ihre Antwort ist zu lang. Kürzen Sie einige Sätze in Ihrer Antwort.

5-iv) Quality assurance methods

Provide information on quality assurance methods to ensure accuracy and quality of information provided [1], if applicable.

	1	2	3	4	5	
subitem not at all important	○	○	◉	○	○	essential

Auswahl löschen

Does your paper address subitem 5-iv?

Copy and paste relevant sections from the manuscript (include quotes in quotation marks "like this" to indicate direct quotes from your manuscript), or elaborate on this item by providing additional information not in the ms, or briefly explain why the item is not applicable/relevant for your study

We utilized previously validated questionnaires for outcome assessment. For one questionnaire, we used a selection of items from a previously validated questionnaire: "Stress-related (dys-)functional and dysfunctional beliefs were assessed using 10 selected items from the short version of the DAS [71]. In total, 9 of these items addressed dysfunctional beliefs, whereas the last item assessed a functional belief. The items were selected based on the extent of to which they fitted to the topic of stress by a group of 3 experts in clinical psychology (including the last author). " The items presented in the training were selected as follows: "The pool of 78 stress-related beliefs was based on the Dysfunctional Attitudes Scale (DAS) [71] and supplemented by additional stress-relevant beliefs developed by a group of experts in clinical psychology."

Ihre Antwort ist zu lang. Kürzen Sie einige Sätze in Ihrer Antwort.

5-v) Ensure replicability by publishing the source code, and/or providing screenshots/screen-capture video, and/or providing flowcharts of the algorithms used

Ensure replicability by publishing the source code, and/or providing screenshots/screen-capture video, and/or providing flowcharts of the algorithms used. Replicability (i.e., other researchers should in principle be able to replicate the study) is a hallmark of scientific reporting.

	1	2	3	4	5	
subitem not at all important	○	○	◉	○	○	essential

Auswahl löschen

Does your paper address subitem 5-v?

Copy and paste relevant sections from the manuscript (include quotes in quotation marks "like this" to indicate direct quotes from your manuscript), or elaborate on this item by providing additional information not in the ms, or briefly explain why the item is not applicable/relevant for your study

This application is still under development. The functionalities of the app are not yet finalized. However, researchers who wish to use the app may contact the authors.

5-vi) Digital preservation

Digital preservation: Provide the URL of the application, but as the intervention is likely to change or disappear over the course of the years; also make sure the intervention is archived (Internet Archive, webcitation.org, and/or publishing the source code or screenshots/videos alongside the article). As pages behind login screens cannot be archived, consider creating demo pages which are accessible without login.

	1	2	3	4	5	
subitem not at all important	○	○	○	◉	○	essential

Auswahl löschen

Ihre Antwort ist zu lang. Kürzen Sie einige Sätze in Ihrer Antwort.

Does your paper address subitem 5-vi?

Copy and paste relevant sections from the manuscript (include quotes in quotation marks "like this" to indicate direct quotes from your manuscript), or elaborate on this item by providing additional information not in the ms, or briefly explain why the item is not applicable/relevant for your study

As this intervention is still under development, it is not yet publicly available.

5-vii) Access

Access: Describe how participants accessed the application, in what setting/context, if they had to pay (or were paid) or not, whether they had to be a member of specific group. If known, describe how participants obtained “access to the platform and Internet” [1]. To ensure access for editors/reviewers/readers, consider to provide a “backdoor” login account or demo mode for reviewers/readers to explore the application (also important for archiving purposes, see vi).

	1	2	3	4	5	
subitem not at all important	○	○	○	◉	○	essential

Auswahl löschen

Does your paper address subitem 5-vii? *

Copy and paste relevant sections from the manuscript (include quotes in quotation marks "like this" to indicate direct quotes from your manuscript), or elaborate on this item by providing additional information not in the ms, or briefly explain why the item is not applicable/relevant for your study

A smartphone with the application was provided to participants during the study. We did not report this specifically, as the present study was a pilot study and all procedures took place in a laboratory. The application is still in under development and not yet intended for wider distribution.

Ihre Antwort ist zu lang. Kürzen Sie einige Sätze in Ihrer Antwort.

5-viii) Mode of delivery, features/functionalities/components of the intervention and comparator, and the theoretical framework

Describe mode of delivery, features/functionalities/components of the intervention and comparator, and the theoretical framework [6] used to design them (instructional strategy [1], behaviour change techniques, persuasive features, etc., see e.g., [7, 8] for terminology). This includes an in-depth description of the content (including where it is coming from and who developed it) [1],” whether [and how] it is tailored to individual circumstances and allows users to track their progress and receive feedback” [6]. This also includes a description of communication delivery channels and – if computer-mediated communication is a component – whether communication was synchronous or asynchronous [6]. It also includes information on presentation strategies [1], including page design principles, average amount of text on pages, presence of hyperlinks to other resources, etc. [1].

	1	2	3	4	5	
subitem not at all important	○	○	○	◉	○	essential

Auswahl löschen

Does your paper address subitem 5-viii? *

Copy and paste relevant sections from the manuscript (include quotes in quotation marks "like this" to indicate direct quotes from your manuscript), or elaborate on this item by providing additional information not in the ms, or briefly explain why the item is not applicable/relevant for your study

Presentation of stress-enhancing and stress-reducing beliefs written statements (60 beliefs randomly selected of 78 beliefs (30 stress-enhancing beliefs and 30 stress-reducing beliefs, randomly selected from a pool of 48 beliefs). 50:50 ratio for the presentation of stress-reducing and stress-enhancing beliefs), pool of 78 stress-related beliefs was based on the Dysfunctional Attitudes Scale (DAS) [71] + additional stress-relevant beliefs developed by a group of experts in clinical psychology. Beliefs were presented in a random order during each training session. Participants were instructed to perform an approach response when presented with a stress-reducing belief and to perform an avoidance response when presented with a stress-enhancing belief." Participants received feedback on whether they had shown the correct response or not.

Ihre Antwort ist zu lang. Kürzen Sie einige Sätze in Ihrer Antwort.

5-ix) Describe use parameters

Describe use parameters (e.g., intended “doses” and optimal timing for use). Clarify what instructions or recommendations were given to the user, e.g., regarding timing, frequency, heaviness of use, if any, or was the intervention used ad libitum.

	1	2	3	4	5	
subitem not at all important	○	○	◉	○	○	essential

Auswahl löschen

Does your paper address subitem 5-ix?

Copy and paste relevant sections from the manuscript (include quotes in quotation marks "like this" to indicate direct quotes from your manuscript), or elaborate on this item by providing additional information not in the ms, or briefly explain why the item is not applicable/relevant for your study

As this application is still under development, we have not yet specified such parameters.

5-x) Clarify the level of human involvement

Clarify the level of human involvement (care providers or health professionals, also technical assistance) in the e-intervention or as co-intervention (detail number and expertise of professionals involved, if any, as well as “type of assistance offered, the timing and frequency of the support, how it is initiated, and the medium by which the assistance is delivered”. It may be necessary to distinguish between the level of human involvement required for the trial, and the level of human involvement required for a routine application outside of a RCT setting (discuss under item 21 – generalizability).

	1	2	3	4	5	
subitem not at all important	○	○	◉	○	○	essential

Auswahl löschen

Ihre Antwort ist zu lang. Kürzen Sie einige Sätze in Ihrer Antwort.

Does your paper address subitem 5-x?

Copy and paste relevant sections from the manuscript (include quotes in quotation marks "like this" to indicate direct quotes from your manuscript), or elaborate on this item by providing additional information not in the ms, or briefly explain why the item is not applicable/relevant for your study

"Participants completed 2 practice trials together with the experimenter and were provided feedback on whether they had shown the correct response. During the training, the experimenter monitored the participants' responses and emotional expressions through a video feed and triggered movements of the stimuli on the smartphone screen via a remote control if the emotion was shown as instructed (similar to a Wizard-of-Oz paradigm [72]; but modified in the sense that participants always knew that the stimuli were moved by the experimenter)."

5-xi) Report any prompts/reminders used

Report any prompts/reminders used: Clarify if there were prompts (letters, emails, phone calls, SMS) to use the application, what triggered them, frequency etc. It may be necessary to distinguish between the level of prompts/reminders required for the trial, and the level of prompts/reminders for a routine application outside of a RCT setting (discuss under item 21 – generalizability).

	1	2	3	4	5	
subitem not at all important	○	○	○	◉	○	essential

Auswahl löschen

Does your paper address subitem 5-xi? *

Copy and paste relevant sections from the manuscript (include quotes in quotation marks "like this" to indicate direct quotes from your manuscript), or elaborate on this item by providing additional information not in the ms, or briefly explain why the item is not applicable/relevant for your study

All of the training sessions were conducted in the laboratory and participants only had access to the intervention there. Thus, it was not necessary to send participants reminders to use the application. Any potential drop-out during the course of the training session is documented in the CONSORT flow chart in Figure 1.

Ihre Antwort ist zu lang. Kürzen Sie einige Sätze in Ihrer Antwort.

5-xii) Describe any co-interventions (incl. training/support)

Describe any co-interventions (incl. training/support): Clearly state any interventions that are provided in addition to the targeted eHealth intervention, as ehealth intervention may not be designed as stand-alone intervention. This includes training sessions and support [1]. It may be necessary to distinguish between the level of training required for the trial, and the level of training for a routine application outside of a RCT setting (discuss under item 21 – generalizability.

	1	2	3	4	5	
subitem not at all important	○	○	○	○	◉	essential

Auswahl löschen

Does your paper address subitem 5-xii? *

Copy and paste relevant sections from the manuscript (include quotes in quotation marks "like this" to indicate direct quotes from your manuscript), or elaborate on this item by providing additional information not in the ms, or briefly explain why the item is not applicable/relevant for your study

As the primary aim of this study was to evaluate the feasibility of our novel training paradigm in a pilot study, participants did not receive any additional interventions. Assessment of the efficacy of the paradigm as a standalone versus blended intervention would take place after the paradigm is finalized.

6a) Completely defined pre-specified primary and secondary outcome measures, including how and when they were assessed

Ihre Antwort ist zu lang. Kürzen Sie einige Sätze in Ihrer Antwort.

Does your paper address CONSORT subitem 6a? *

Copy and paste relevant sections from the manuscript (include quotes in quotation marks "like this" to indicate direct quotes from your manuscript), or elaborate on this item by providing additional information not in the ms, or briefly explain why the item is not applicable/relevant for your study

Validated, commonly fulfilling quality criteria. Primary outcome: Feasibility (technical problems = ≥95% of trials without technical difficulties; adherence to the intervention = completion of ≥50%; feasibility of study design = ≥75% completing T3). Usability: 4 (a) intelligibility of instructions, (b) perceived ease of displaying emotions, (c) distance from dysfunctional beliefs, and (d) approach of functional beliefs). Acceptability: 2 items, (a) modification of stress-related beliefs and (b) recommendation of training). 4 clinical variables: Subjective stress (German version of the PSS-10 [67]), agreement with/perceived helpfulness of stress-related beliefs (10 items from DAS [71]; 9 dysfunctional and 1 functional belief), emotion regulation skills (ERSQ; German version: [74]), and depressive symptoms (German version of CES-D [80]). Assessed at T1, T2, and

6a-i) Online questionnaires: describe if they were validated for online use and apply CHERRIES items to describe how the questionnaires were designed/deployed

If outcomes were obtained through online questionnaires, describe if they were validated for online use and apply CHERRIES items to describe how the questionnaires were designed/deployed [9].

	1	2	3	4	5	
subitem not at all important	○	○	◉	○	○	essential

Auswahl löschen

Does your paper address subitem 6a-i?

Copy and paste relevant sections from manuscript text

The questionnaires were not validated for online use.

Ihre Antwort ist zu lang. Kürzen Sie einige Sätze in Ihrer Antwort.

6a-ii) Describe whether and how "use" (including intensity of use/dosage) was defined/measured/monitored

Describe whether and how "use" (including intensity of use/dosage) was defined/measured/monitored (logins, logfile analysis, etc.). Use/adoption metrics are important process outcomes that should be reported in any ehealth trial.

	1	2	3	4	5	
subitem not at all important	○	○	○	◉	○	essential

Auswahl löschen

Does your paper address subitem 6a-ii?

Copy and paste relevant sections from manuscript text

A research assistant supervised each training session and instructed participants on how to complete the training.

6a-iii) Describe whether, how, and when qualitative feedback from participants was obtained

Describe whether, how, and when qualitative feedback from participants was obtained (e.g., through emails, feedback forms, interviews, focus groups).

	1	2	3	4	5	
subitem not at all important	○	○	○	◉	○	essential

Auswahl löschen

Does your paper address subitem 6a-iii?

Copy and paste relevant sections from manuscript text

Qualitative feedback was obtained from participants after the last training session and via the web-based questionnaire tool that was also used for other assessments. The feedback [illegible]

Ihre Antwort ist zu lang. Kürzen Sie einige Sätze in Ihrer Antwort.

6b) Any changes to trial outcomes after the trial commenced, with reasons

Does your paper address CONSORT subitem 6b? *

Copy and paste relevant sections from the manuscript (include quotes in quotation marks "like this" to indicate direct quotes from your manuscript), or elaborate on this item by providing additional information not in the ms, or briefly explain why the item is not applicable/relevant for your study

No changes to the outcome measures were made during the trial.

7a) How sample size was determined

NPT: When applicable, details of whether and how the clustering by care provides or centers was addressed

7a-i) Describe whether and how expected attrition was taken into account when calculating the sample size

Describe whether and how expected attrition was taken into account when calculating the sample size.

	1	2	3	4	5	
subitem not at all important	○	○	○	○	◉	essential

Auswahl löschen

Does your paper address subitem 7a-i?

Copy and paste relevant sections from manuscript title (include quotes in quotation marks "like this" to indicate direct quotes from your manuscript), or elaborate on this item by providing additional information not in the ms, or briefly explain why the item is not applicable/relevant for your study

"As recommended by Whitehead and colleagues [70], we chose a sample size of n = 10 for each treatment arm. Eligible participants were randomly allocated to 1 of the 3 study conditions (eAAMT-SP, swipe control group, and inactive control group)."

Ihre Antwort ist zu lang. Kürzen Sie einige Sätze in Ihrer Antwort.

7b) When applicable, explanation of any interim analyses and stopping guidelines

Does your paper address CONSORT subitem 7b? *

Copy and paste relevant sections from the manuscript (include quotes in quotation marks "like this" to indicate direct quotes from your manuscript), or elaborate on this item by providing additional information not in the ms, or briefly explain why the item is not applicable/relevant for your study

No interim analyses were made, data collection was stopped after the target sample size of N = 30 was reached.

8a) Method used to generate the random allocation sequence

NPT: When applicable, how care providers were allocated to each trial group

Does your paper address CONSORT subitem 8a? *

Copy and paste relevant sections from the manuscript (include quotes in quotation marks "like this" to indicate direct quotes from your manuscript), or elaborate on this item by providing additional information not in the ms, or briefly explain why the item is not applicable/relevant for your study

"Eligible participants were randomly allocated to 1 of the 3 study conditions (eAAMT-SP, swipe control group, and inactive control group). For this purpose, we used block randomization (according to random numbers generated in Microsoft Excel [Microsoft Corp]) with a block size of 8. Randomization was conducted by a research assistant who was otherwise not involved in the study. An overview of the participant flow is shown in Figure 1."

8b) Type of randomisation; details of any restriction (such as blocking and block size)

Ihre Antwort ist zu lang. Kürzen Sie einige Sätze in Ihrer Antwort.

Does your paper address CONSORT subitem 8b? *

Copy and paste relevant sections from the manuscript (include quotes in quotation marks "like this" to indicate direct quotes from your manuscript), or elaborate on this item by providing additional information not in the ms, or briefly explain why the item is not applicable/relevant for your study

"For this purpose, we used block randomization (according to random numbers generated in Microsoft Excel [Microsoft Corp]) with a block size of 8."

9) Mechanism used to implement the random allocation sequence (such as sequentially numbered containers), describing any steps taken to conceal the sequence until interventions were assigned

Does your paper address CONSORT subitem 9? *

Copy and paste relevant sections from the manuscript (include quotes in quotation marks "like this" to indicate direct quotes from your manuscript), or elaborate on this item by providing additional information not in the ms, or briefly explain why the item is not applicable/relevant for your study

"Randomization was conducted by a research assistant who was otherwise not involved in the study. An overview of the participant flow is shown in Figure 1."

10) Who generated the random allocation sequence, who enrolled participants, and who assigned participants to interventions

Does your paper address CONSORT subitem 10? *

Copy and paste relevant sections from the manuscript (include quotes in quotation marks "like this" to indicate direct quotes from your manuscript), or elaborate on this item by providing additional information not in the ms, or briefly explain why the item is not applicable/relevant for your study

"Randomization was conducted by a research assistant who was otherwise not involved in the study. An overview of the participant flow is shown in Figure 1."

Ihre Antwort ist zu lang. Kürzen Sie einige Sätze in Ihrer Antwort.

11a) If done, who was blinded after assignment to interventions (for example, participants, care providers, those assessing outcomes) and how

NPT: Whether or not administering co-interventions were blinded to group assignment

11a-i) Specify who was blinded, and who wasn’t

Specify who was blinded, and who wasn’t. Usually, in web-based trials it is not possible to blind the participants [1, 3] (this should be clearly acknowledged), but it may be possible to blind outcome assessors, those doing data analysis or those administering co-interventions (if any).

	1	2	3	4	5	
subitem not at all important	○	○	○	◉	○	essential

Auswahl löschen

Does your paper address subitem 11a-i? *

Copy and paste relevant sections from the manuscript (include quotes in quotation marks "like this" to indicate direct quotes from your manuscript), or elaborate on this item by providing additional information not in the ms, or briefly explain why the item is not applicable/relevant for your study

"Participants who were allocated to either the eAAMT-SP (experimental condition) or the swipe control condition were blinded to whether they had received the experimental intervention or the active control intervention. Participants who were allocated to the inactive control group were aware that they had not received any intervention. The study personnel delivering the interventions were not blinded to the group allocation that the participants received"

Ihre Antwort ist zu lang. Kürzen Sie einige Sätze in Ihrer Antwort.

11a-ii) Discuss e.g., whether participants knew which intervention was the "intervention of interest" and which one was the "comparator"

Informed consent procedures (4a-ii) can create biases and certain expectations - discuss e.g., whether participants knew which intervention was the "intervention of interest" and which one was the "comparator".

	1	2	3	4	5	
subitem not at all important	○	○	○	◉	○	essential

Auswahl löschen

Does your paper address subitem 11a-ii?

Copy and paste relevant sections from the manuscript (include quotes in quotation marks "like this" to indicate direct quotes from your manuscript), or elaborate on this item by providing additional information not in the ms, or briefly explain why the item is not applicable/relevant for your study

Participants allocated to either the eAAMT-SP or the swipe control condition were not aware whether they had been assigned to the experimental condition or the active control

11b) If relevant, description of the similarity of interventions

(this item is usually not relevant for ehealth trials as it refers to similarity of a placebo or sham intervention to a active medication/intervention)

Ihre Antwort ist zu lang. Kürzen Sie einige Sätze in Ihrer Antwort.

Does your paper address CONSORT subitem 11b? *

Copy and paste relevant sections from the manuscript (include quotes in quotation marks "like this" to indicate direct quotes from your manuscript), or elaborate on this item by providing additional information not in the ms, or briefly explain why the item is not applicable/relevant for your study

"In the eAAMT-SP condition, participants were instructed to display sadness in response to stress-enhancing beliefs. More specifically, they were invited to display a sad facial expression, take a deep breath, tilt their head forward, and say “I will let go of this belief.". In response to stress-reducing beliefs, participants were instructed to display either joy, relaxation, or love on the first day of training; excitement, tranquillity, or gratitude on the second day; happiness, resolve, or contentment on the third day; and courage, confidence, or pride on the last day of training." "In the swipe control condition, participants were instructed to swipe stress-enhancing beliefs away from themselves (from bottom to top) and stress-reducing beliefs towards themselves (from top to bottom) on the smartphone

12a) Statistical methods used to compare groups for primary and secondary outcomes

NPT: When applicable, details of whether and how the clustering by care providers or centers was addressed

Does your paper address CONSORT subitem 12a? *

Copy and paste relevant sections from the manuscript (include quotes in quotation marks "like this" to indicate direct quotes from your manuscript), or elaborate on this item by providing additional information not in the ms, or briefly explain why the item is not applicable/relevant for your study

Feasibility: Comparison of indicators of feasibility between the eAAMT-SP and swipe control conditions (descriptively and using t-tests). Clinical potential of eAAMT-SP: Hedge’s g [86] effect sizes for descriptive comparisons across study conditions. "To explore whether differences across conditions would meet the criteria for statistical significance in spite of the small sample size, we computed a 95% bootstrap confidence CI (calculated with using n = 1000 iterations) and tested differences across conditions regarding primary and secondary clinical outcomes with 2-way analyses of variance (ANOVAs). In these analyses, the study condition was included as the between-factor, and time was included as the within-factor." "The Hedge’s g was calculated using the rstatix-package [90]. The ANOVA was conducted using the afex- package [91]."

Ihre Antwort ist zu lang. Kürzen Sie einige Sätze in Ihrer Antwort.

12a-i) Imputation techniques to deal with attrition / missing values

Imputation techniques to deal with attrition / missing values: Not all participants will use the intervention/comparator as intended and attrition is typically high in ehealth trials. Specify how participants who did not use the application or dropped out from the trial were treated in the statistical analysis (a complete case analysis is strongly discouraged, and simple imputation techniques such as LOCF may also be problematic [4]).

	1	2	3	4	5	
subitem not at all important	○	○	○	○	◉	essential

Auswahl löschen

Does your paper address subitem 12a-i? *

Copy and paste relevant sections from the manuscript (include quotes in quotation marks "like this" to indicate direct quotes from your manuscript), or elaborate on this item by providing additional information not in the ms, or briefly explain why the item is not applicable/relevant for your study

"The ANOVA was evaluatedconducted using both a per-protocol approach and the ITT principle. For the latter, missing data were imputed using a multiple-chained equations approach [88] with m = 20 imputations. Missing values in clinical outcome variables were imputed from the available data of these variables (PSS-10, DAS-A, DAS-H, ERSQ, and CES-D)." "Multiple imputation was carried out using the mice- package [92], and the ANOVA for the ITT analysis was calculated conducted using the miceadds- package [93]."

12b) Methods for additional analyses, such as subgroup analyses and adjusted analyses

Does your paper address CONSORT subitem 12b? *

Copy and paste relevant sections from the manuscript (include quotes in quotation marks "like this" to indicate direct quotes from your manuscript), or elaborate on this item by providing additional information not in the ms, or briefly explain why the item is not applicable/relevant for your study

We did not conduct any subgroup analyses.

Ihre Antwort ist zu lang. Kürzen Sie einige Sätze in Ihrer Antwort.

X26) REB/IRB Approval and Ethical Considerations [recommended as subheading under "Methods"] (not a CONSORT item)

X26-i) Comment on ethics committee approval

	1	2	3	4	5	
subitem not at all important	○	○	○	○	◉	essential

Auswahl löschen

Does your paper address subitem X26-i?

Copy and paste relevant sections from the manuscript (include quotes in quotation marks "like this" to indicate direct quotes from your manuscript), or elaborate on this item by providing additional information not in the ms, or briefly explain why the item is not applicable/relevant for your study

"The study was conducted in accordance with ethical standards as defined in the Declaration of Helsinki and was approved by the German Psychological Society ethics committee (BerkingMatthias2020-09-10AM)."

x26-ii) Outline informed consent procedures

Outline informed consent procedures e.g., if consent was obtained offline or online (how? Checkbox, etc.?), and what information was provided (see 4a-ii). See [6] for some items to be included in informed consent documents.

	1	2	3	4	5	
subitem not at all important	○	○	○	○	◉	essential

Auswahl löschen

Ihre Antwort ist zu lang. Kürzen Sie einige Sätze in Ihrer Antwort.

Does your paper address subitem X26-ii?

Copy and paste relevant sections from the manuscript (include quotes in quotation marks "like this" to indicate direct quotes from your manuscript), or elaborate on this item by providing additional information not in the ms, or briefly explain why the item is not applicable/relevant for your study

"Participants provided their written informed consent before filling out the web-based screening questionnaire."

X26-iii) Safety and security procedures

Safety and security procedures, incl. privacy considerations, and any steps taken to reduce the likelihood or detection of harm (e.g., education and training, availability of a hotline)

	1	2	3	4	5	
subitem not at all important	○	○	○	◉	○	essential

Auswahl löschen

Does your paper address subitem X26-iii?

Copy and paste relevant sections from the manuscript (include quotes in quotation marks "like this" to indicate direct quotes from your manuscript), or elaborate on this item by providing additional information not in the ms, or briefly explain why the item is not applicable/relevant for your study

Data were only transferred over a secure network connection. Participants with any grave mental illness of current suicidal ideation were not considered for the participation in the study. During the training, a research assistant was present at all times.

RESULTS

Ihre Antwort ist zu lang. Kürzen Sie einige Sätze in Ihrer Antwort.

13a) For each group, the numbers of participants who were randomly assigned, received intended treatment, and were analysed for the primary outcome
NPT: The number of care providers or centers performing the intervention in each group and the number of patients treated by each care provider in each center

Does your paper address CONSORT subitem 13a? *
Copy and paste relevant sections from the manuscript (include quotes in quotation marks "like this" to indicate direct quotes from your manuscript), or elaborate on this item by providing additional information not in the ms, or briefly explain why the item is not applicable/relevant for your study

Figure 1 details the participant flow including all participants who were recruited, screened, randomized, completed the treatment, and were analyzed.

13b) For each group, losses and exclusions after randomisation, together with reasons

Does your paper address CONSORT subitem 13b? (NOTE: Preferably, this is shown in a CONSORT flow diagram) *
Copy and paste relevant sections from the manuscript (include quotes in quotation marks "like this" to indicate direct quotes from your manuscript), or elaborate on this item by providing additional information not in the ms, or briefly explain why the item is not applicable/relevant for your study

Figure 1 details the losses and exclusion for each group.

Ihre Antwort ist zu lang. Kürzen Sie einige Sätze in Ihrer Antwort.

13b-i) Attrition diagram

Strongly recommended: An attrition diagram (e.g., proportion of participants still logging in or using the intervention/comparator in each group plotted over time, similar to a survival curve) or other figures or tables demonstrating usage/dose/engagement.

	1	2	3	4	5	
subitem not at all important	○	○	○	◉	○	essential

Auswahl löschen

Does your paper address subitem 13b-i?

Copy and paste relevant sections from the manuscript or cite the figure number if applicable (include quotes in quotation marks "like this" to indicate direct quotes from your manuscript), or elaborate on this item by providing additional information not in the ms, or briefly explain why the item is not applicable/relevant for your study

As the participants only received the intervention for the duration of the study, they did not continue to use the application after the study was completed.

14a) Dates defining the periods of recruitment and follow-up

Does your paper address CONSORT subitem 14a? *

Copy and paste relevant sections from the manuscript (include quotes in quotation marks "like this" to indicate direct quotes from your manuscript), or elaborate on this item by providing additional information not in the ms, or briefly explain why the item is not applicable/relevant for your study

"Recruiting took place in Erlangen, Germany, via posts on social networks, email newsletters of the Friedrich-Alexander-Universität Erlangen-Nürnberg, and flyers posted in public places between July 2020 and May 2022."

Ihre Antwort ist zu lang. Kürzen Sie einige Sätze in Ihrer Antwort.

14a-i) Indicate if critical “secular events” fell into the study period

Indicate if critical “secular events” fell into the study period, e.g., significant changes in Internet resources available or “changes in computer hardware or Internet delivery resources”

	1	2	3	4	5	
subitem not at all important	○	○	◉	○	○	essential

Auswahl löschen

Does your paper address subitem 14a-i?

Copy and paste relevant sections from the manuscript (include quotes in quotation marks "like this" to indicate direct quotes from your manuscript), or elaborate on this item by providing additional information not in the ms, or briefly explain why the item is not applicable/relevant for your study

Participants only interacted with the application while they were in the laboratory for the study. Technical events that would lead to delay or termination of the training were evaluated as part of the feasibility evaluation: "Technical problems were evaluated by assessing whether ≥95% of the study trials were completed without technical difficulties that would lead to termination of, or interruption of, or delays of >5 minutes in the study trial." Regarding non-technical events, recruitment was conducted during the COVID-19 pandemic, which may have affected recruitment rates.

14b) Why the trial ended or was stopped (early)

Does your paper address CONSORT subitem 14b? *

Copy and paste relevant sections from the manuscript (include quotes in quotation marks "like this" to indicate direct quotes from your manuscript), or elaborate on this item by providing additional information not in the ms, or briefly explain why the item is not applicable/relevant for your study

Recruitment was stopped after the target sample size of N = 30 was reached.

Ihre Antwort ist zu lang. Kürzen Sie einige Sätze in Ihrer Antwort.

15) A table showing baseline demographic and clinical characteristics for each group
NPT: When applicable, a description of care providers (case volume, qualification, expertise, etc.) and centers (volume) in each group

Does your paper address CONSORT subitem 15? *

Copy and paste relevant sections from the manuscript (include quotes in quotation marks "like this" to indicate direct quotes from your manuscript), or elaborate on this item by providing additional information not in the ms, or briefly explain why the item is not applicable/relevant for your study

Table 1 details the demographic characteristics for the three experimental groups (gender, age, education level, occupation, study period, field of study, marital status, former psychotherapy, and current psychotherapy).

15-i) Report demographics associated with digital divide issues

In ehealth trials it is particularly important to report demographics associated with digital divide issues, such as age, education, gender, social-economic status, computer/Internet /ehealth literacy of the participants, if known.

	1	2	3	4	5	
subitem not at all important	○	○	○	◉	○	essential

Auswahl löschen

Does your paper address subitem 15-i? *

Copy and paste relevant sections from the manuscript (include quotes in quotation marks "like this" to indicate direct quotes from your manuscript), or elaborate on this item by providing additional information not in the ms, or briefly explain why the item is not applicable/relevant for your study

This does not apply to the current study, as participants were recruited offline and were provided with a smartphone for the training in the laboratory.

Ihre Antwort ist zu lang. Kürzen Sie einige Sätze in Ihrer Antwort.

16) For each group, number of participants (denominator) included in each analysis and whether the analysis was by original assigned groups

16-i) Report multiple “denominators” and provide definitions

Report multiple “denominators” and provide definitions: Report N’s (and effect sizes) “across a range of study participation [and use] thresholds” [1], e.g., N exposed, N consented, N used more than x times, N used more than y weeks, N participants “used” the intervention/comparator at specific pre-defined time points of interest (in absolute and relative numbers per group). Always clearly define “use” of the intervention.

	1	2	3	4	5	
subitem not at all important	○	◉	○	○	○	essential

Auswahl löschen

Does your paper address subitem 16-i? *

Copy and paste relevant sections from the manuscript (include quotes in quotation marks "like this" to indicate direct quotes from your manuscript), or elaborate on this item by providing additional information not in the ms, or briefly explain why the item is not applicable/relevant for your study

This is not applicable for the current study, as all participants only used (= interacted with) the intervention while they were in the laboratory for completing the training. Thus, all information regarding usage is equal to the information on the participant flow in Figure 1.

16-ii) Primary analysis should be intent-to-treat

Primary analysis should be intent-to-treat, secondary analyses could include comparing only “users”, with the appropriate caveats that this is no longer a randomized sample (see 18-i).

	1	2	3	4	5	
subitem not at all important	○	○	◉	○	○	essential

Auswahl löschen

Ihre Antwort ist zu lang. Kürzen Sie einige Sätze in Ihrer Antwort.

Does your paper address subitem 16-ii?

Copy and paste relevant sections from the manuscript (include quotes in quotation marks "like this" to indicate direct quotes from your manuscript), or elaborate on this item by providing additional information not in the ms, or briefly explain why the item is not applicable/relevant for your study

We reported the results of the per-protocol analyses, as data was missing at random and the results of the intention-to-treat analyses did not differ from those of the per protocol analyses.

17a) For each primary and secondary outcome, results for each group, and the estimated effect size and its precision (such as 95% confidence interval)

Does your paper address CONSORT subitem 17a? *

Copy and paste relevant sections from the manuscript (include quotes in quotation marks "like this" to indicate direct quotes from your manuscript), or elaborate on this item by providing additional information not in the ms, or briefly explain why the item is not applicable/relevant for your study

Feasibility: The criteria for technical feasibility and feasibility of the study design were not met, while the criterion for intervention completion was met. (for further information see manuscript, heading "Feasibility", "Primary Clinical Outcome", and "Secondary Clinical Outcome")

Ihre Antwort ist zu lang. Kürzen Sie einige Sätze in Ihrer Antwort.

17a-i) Presentation of process outcomes such as metrics of use and intensity of use

In addition to primary/secondary (clinical) outcomes, the presentation of process outcomes such as metrics of use and intensity of use (dose, exposure) and their operational definitions is critical. This does not only refer to metrics of attrition (13-b) (often a binary variable), but also to more continuous exposure metrics such as “average session length”. These must be accompanied by a technical description how a metric like a “session” is defined (e.g., timeout after idle time) [1] (report under item 6a).

	1	2	3	4	5	
subitem not at all important	○	○	○	◉	○	essential

Auswahl löschen

Does your paper address subitem 17a-i?

Copy and paste relevant sections from the manuscript (include quotes in quotation marks "like this" to indicate direct quotes from your manuscript), or elaborate on this item by providing additional information not in the ms, or briefly explain why the item is not applicable/relevant for your study

This item does not apply for the current study, as all usage of the application only took place during the training in the laboratory and all participants who had completed the training had used the application to the full extent.

17b) For binary outcomes, presentation of both absolute and relative effect sizes is recommended

Does your paper address CONSORT subitem 17b? *

Copy and paste relevant sections from the manuscript (include quotes in quotation marks "like this" to indicate direct quotes from your manuscript), or elaborate on this item by providing additional information not in the ms, or briefly explain why the item is not applicable/relevant for your study

This item is not applicable, as we did not include binary primary outcomes.

Ihre Antwort ist zu lang. Kürzen Sie einige Sätze in Ihrer Antwort.

18) Results of any other analyses performed, including subgroup analyses and adjusted analyses, distinguishing pre-specified from exploratory

Does your paper address CONSORT subitem 18? *

Copy and paste relevant sections from the manuscript (include quotes in quotation marks "like this" to indicate direct quotes from your manuscript), or elaborate on this item by providing additional information not in the ms, or briefly explain why the item is not applicable/relevant for your study

We conducted exploratory ANOVAs to compare the effects between groups. As the results are too long to be presented in this checklist, please refer to see manuscript, heading "Exploratory ANOVAs"

18-i) Subgroup analysis of comparing only users

A subgroup analysis of comparing only users is not uncommon in ehealth trials, but if done, it must be stressed that this is a self-selected sample and no longer an unbiased sample from a randomized trial (see 16-iii).

	1	2	3	4	5	
subitem not at all important	○	○	◉	○	○	essential

Auswahl löschen

Does your paper address subitem 18-i?

Copy and paste relevant sections from the manuscript (include quotes in quotation marks "like this" to indicate direct quotes from your manuscript), or elaborate on this item by providing additional information not in the ms, or briefly explain why the item is not applicable/relevant for your study

This is not applicable for the current study, as we did not include such a sample.

19) All important harms or unintended effects in each group

Ihre Antwort ist zu lang. Kürzen Sie einige Sätze in Ihrer Antwort.

Does your paper address CONSORT subitem 19? *

Copy and paste relevant sections from the manuscript (include quotes in quotation marks "like this" to indicate direct quotes from your manuscript), or elaborate on this item by providing additional information not in the ms, or briefly explain why the item is not applicable/relevant for your study

No harms or unintended effects occurred in any group.

19-i) Include privacy breaches, technical problems

Include privacy breaches, technical problems. This does not only include physical “harm” to participants, but also incidents such as perceived or real privacy breaches [1], technical problems, and other unexpected/unintended incidents. “Unintended effects” also includes unintended positive effects [2].

	1	2	3	4	5	
subitem not at all important	○	○	○	◉	○	essential

Auswahl löschen

Does your paper address subitem 19-i?

Copy and paste relevant sections from the manuscript (include quotes in quotation marks "like this" to indicate direct quotes from your manuscript), or elaborate on this item by providing additional information not in the ms, or briefly explain why the item is not applicable/relevant for your study

We did not assess any additional technical difficulties beyond technical problems that would lead to delays in or termination of the training appointment. The data generated in the application during the training was stored locally on the smartphone.

Ihre Antwort ist zu lang. Kürzen Sie einige Sätze in Ihrer Antwort.

19-ii) Include qualitative feedback from participants or observations from staff/researchers

Include qualitative feedback from participants or observations from staff/researchers, if available, on strengths and shortcomings of the application, especially if they point to unintended/unexpected effects or uses. This includes (if available) reasons for why people did or did not use the application as intended by the developers.

	1	2	3	4	5	
subitem not at all important	○	○	◉	○	○	essential

Auswahl löschen

Does your paper address subitem 19-ii?

Copy and paste relevant sections from the manuscript (include quotes in quotation marks "like this" to indicate direct quotes from your manuscript), or elaborate on this item by providing additional information not in the ms, or briefly explain why the item is not applicable/relevant for your study

We did collect qualitative feedback from participants after completion of the training, but we did not evaluate it in this manuscript as it was not the focus of the research question for the current manuscript.

DISCUSSION

22) Interpretation consistent with results, balancing benefits and harms, and considering other relevant evidence

NPT: In addition, take into account the choice of the comparator, lack of or partial blinding, and unequal expertise of care providers or centers in each group

Ihre Antwort ist zu lang. Kürzen Sie einige Sätze in Ihrer Antwort.

22-i) Restate study questions and summarize the answers suggested by the data, starting with primary outcomes and process outcomes (use)

Restate study questions and summarize the answers suggested by the data, starting with primary outcomes and process outcomes (use).

	1	2	3	4	5	
subitem not at all important	○	○	○	○	◉	essential

Auswahl löschen

Does your paper address subitem 22-i? *

Copy and paste relevant sections from the manuscript (include quotes in quotation marks "like this" to indicate direct quotes from your manuscript), or elaborate on this item by providing additional information not in the ms, or briefly explain why the item is not applicable/relevant for your study

"The primary goal of this pilot study was to evaluate the feasibility of a smartphone-based AAMT that utilizes uses expressions of sadness and positive emotions to move stress-enhancing beliefs away from oneself and stress-reducing beliefs towards oneself. Secondly, we aimed to explore the therapeutic potential of the new eAAMT-SP intervention by assessing its effects on the clinical outcomes of perceived stress, agreement with and perceived helpfulness of dysfunctional beliefs, emotion regulation, and depressive symptoms. For this purpose, we compared the eAAMT-SP condition with both an active and an inactive control condition in a sample of n = 30 participants with from elevated

22-ii) Highlight unanswered new questions, suggest future research

Highlight unanswered new questions, suggest future research.

	1	2	3	4	5	
subitem not at all important	○	○	○	○	◉	essential

Auswahl löschen

Ihre Antwort ist zu lang. Kürzen Sie einige Sätze in Ihrer Antwort.

Does your paper address subitem 22-ii?

Copy and paste relevant sections from the manuscript (include quotes in quotation marks "like this" to indicate direct quotes from your manuscript), or elaborate on this item by providing additional information not in the ms, or briefly explain why the item is not applicable/relevant for your study

Replication of present findings in a randomized clinical trial sufficiently powered to allow for confirmatory testing with representative sample, inclusion of comparison with a gold -standard intervention, compare the efficacy of all possible combinations of (a1) using emotions versus finger movements to move stimuli and of (b2) utilizing using stress-related beliefs versus pictures of specific objects cueing stress, inclusion of not only self-report measures, assessment of long-term effects, assessment of effects in clinical

20) Trial limitations, addressing sources of potential bias, imprecision, and, if relevant, multiplicity of analyses

20-i) Typical limitations in ehealth trials

Typical limitations in ehealth trials: Participants in ehealth trials are rarely blinded. Ehealth trials often look at a multiplicity of outcomes, increasing risk for a Type I error. Discuss biases due to non-use of the intervention/usability issues, biases through informed consent procedures, unexpected events.

	1	2	3	4	5	
subitem not at all important	○	○	○	○	◉	essential

Auswahl löschen

Ihre Antwort ist zu lang. Kürzen Sie einige Sätze in Ihrer Antwort.

Does your paper address subitem 20-i? *

Copy and paste relevant sections from the manuscript (include quotes in quotation marks "like this" to indicate direct quotes from your manuscript), or elaborate on this item by providing additional information not in the ms, or briefly explain why the item is not applicable/relevant for your study

Small sample size and (not powered to perform confirmatory testing), short training duration, study use of both the display of emotions to move stimuli (instead of joystick or swipe movements) and beliefs as stimuli (instead of pictures of real objects), use of only self-report measures, sample of only university students (not representative), recruitment took place over a period of 2 years, which was due to slow recruitment during the COVID-19 pandemic (potential cohort effects may be present), no comparison with gold-standard stress interventions

21) Generalisability (external validity, applicability) of the trial findings

NPT: External validity of the trial findings according to the intervention, comparators, patients, and care providers or centers involved in the trial

21-i) Generalizability to other populations

Generalizability to other populations: In particular, discuss generalizability to a general Internet population, outside of a RCT setting, and general patient population, including applicability of the study results for other organizations

	1	2	3	4	5	
subitem not at all important	○	○	○	◉	○	essential

Auswahl löschen

Does your paper address subitem 21-i?

Copy and paste relevant sections from the manuscript (include quotes in quotation marks "like this" to indicate direct quotes from your manuscript), or elaborate on this item by providing additional information not in the ms, or briefly explain why the item is not applicable/relevant for your study

We discussed the issue of generalizability as part of the limitations.

Ihre Antwort ist zu lang. Kürzen Sie einige Sätze in Ihrer Antwort.

21-ii) Discuss if there were elements in the RCT that would be different in a routine application setting

Discuss if there were elements in the RCT that would be different in a routine application setting (e.g., prompts/reminders, more human involvement, training sessions or other co-interventions) and what impact the omission of these elements could have on use, adoption, or outcomes if the intervention is applied outside of a RCT setting.

	1	2	3	4	5	
subitem not at all important	○	○	○	◉	○	essential

Auswahl löschen

Does your paper address subitem 21-ii?

Copy and paste relevant sections from the manuscript (include quotes in quotation marks "like this" to indicate direct quotes from your manuscript), or elaborate on this item by providing additional information not in the ms, or briefly explain why the item is not applicable/relevant for your study

We did not address this topic as the aim of this paper was to evaluate the feasbility of the intervention and it is not finalized yet. After further development, the intervention will need to be evaluated in a fully-powered RCT.

OTHER INFORMATION

23) Registration number and name of trial registry

Does your paper address CONSORT subitem 23? *

Copy and paste relevant sections from the manuscript (include quotes in quotation marks "like this" to indicate direct quotes from your manuscript), or elaborate on this item by providing additional information not in the ms, or briefly explain why the item is not applicable/relevant for your study

German Clinical Trials Registry DRKS00023007

Ihre Antwort ist zu lang. Kürzen Sie einige Sätze in Ihrer Antwort.

24) Where the full trial protocol can be accessed, if available

Does your paper address CONSORT subitem 24? *

Cite a Multimedia Appendix, other reference, or copy and paste relevant sections from the manuscript (include quotes in quotation marks "like this" to indicate direct quotes from your manuscript), or elaborate on this item by providing additional information not in the ms, or briefly explain why the item is not applicable/relevant for your study

The study protocol has been already published: M. Keinert, B. M. Eskofier, B. W. Schuller, S. Böhme, and M. Berking, “Evaluating the feasibility and exploring the efficacy of an emotion-based approach-avoidance modification training (eAAMT) in the context of perceived stress in an adult sample — protocol of a parallel randomized controlled pilot study,” Pilot Feasibility Stud, vol. 9, no. 1, p. 155, Sep. 2023, doi:

25) Sources of funding and other support (such as supply of drugs), role of funders

Does your paper address CONSORT subitem 25? *

Copy and paste relevant sections from the manuscript (include quotes in quotation marks "like this" to indicate direct quotes from your manuscript), or elaborate on this item by providing additional information not in the ms, or briefly explain why the item is not applicable/relevant for your study

"This study is part of the “Optimierung von Apps zur Stärkung der psychischen Gesundheit” [(Optimizing Apps to Strengthen Mental Health]) research project, which is part of the Bavarian Research Association on Healthy Use of Digital Technologies and Media (ForDigitHealth) and is funded by the Bavarian State Ministry of Science and the Arts.
This work was (partly) funded by the Deutsche Forschungsgemeinschaft (DFG , German

X27) Conflicts of Interest (not a CONSORT item)

Ihre Antwort ist zu lang. Kürzen Sie einige Sätze in Ihrer Antwort.

X27-i) State the relation of the study team towards the system being evaluated

In addition to the usual declaration of interests (financial or otherwise), also state the relation of the study team towards the system being evaluated, i.e., state if the authors/evaluators are distinct from or identical with the developers/sponsors of the intervention.

	1	2	3	4	5	
subitem not at all important	○	○	○	○	◉	essential

Auswahl löschen

Does your paper address subitem X27-i?

Copy and paste relevant sections from the manuscript (include quotes in quotation marks "like this" to indicate direct quotes from your manuscript), or elaborate on this item by providing additional information not in the ms, or briefly explain why the item is not applicable/relevant for your study

We have no conflicts of interest to declare.

About the CONSORT EHEALTH checklist

As a result of using this checklist, did you make changes in your manuscript? *

- ○ yes, major changes
- ◉ yes, minor changes
- ○ no

What were the most important changes you made as a result of using this checklist?

Ihre Antwort ist zu lang. Kürzen Sie einige Sätze in Ihrer Antwort.

How much time did you spend on going through the checklist INCLUDING making changes in your manuscript *

I have spent about seven hours completing the checklist.

As a result of using this checklist, do you think your manuscript has improved? *

- (x) yes
- ( ) no
- ( ) Sonstiges:

Would you like to become involved in the CONSORT EHEALTH group?

This would involve for example becoming involved in participating in a workshop and writing an "Explanation and Elaboration" document

- ( ) yes
- (x) no
- ( ) Sonstiges:

Auswahl löschen

Any other comments or questions on CONSORT EHEALTH

Meine Antwort

Ihre Antwort ist zu lang. Kürzen Sie einige Sätze in Ihrer Antwort.

STOP - Save this form as PDF before you click submit

To generate a record that you filled in this form, we recommend to generate a PDF of this page (on a Mac, simply select "print" and then select "print as PDF") before you submit it.

When you submit your (revised) paper to JMIR, please upload the PDF as supplementary file.

Don't worry if some text in the textboxes is cut off, as we still have the complete information in our database. Thank you!

Final step: Click submit !

Click submit so we have your answers in our database!

Senden

Alle Eingaben löschen

Geben Sie niemals Passwörter über Google Formulare weiter.

Dieser Inhalt wurde nicht von Google erstellt und wird von Google auch nicht unterstützt. Missbrauch melden - Nutzungsbedingungen - Datenschutzerklärung

Google Formulare

Ihre Antwort ist zu lang. Kürzen Sie einige Sätze in Ihrer Antwort.
